# Gearing up for action: Attentive tracking dynamically tunes sensory and motor oscillations in the *alpha* and *beta* band^☆^

**DOI:** 10.1016/j.neuroimage.2013.04.120

**Published:** 2013-11-15

**Authors:** Heng-Ru May Tan, Hartmut Leuthold, Joachim Gross

**Affiliations:** a Centre for Cognitive Neuroimaging (CCNi), Institute of Neuroscience and Psychology, College of Science and Engineering & College of Medical, Veterinary and Life Sciences, University of Glasgow, 58 Hillhead Street, Glasgow G12 8QB, UK; b Department of Psychology, Eberhard Karls Universität Tübingen, Schleichstr. 4, 72076 Tübingen, Germany

**Keywords:** *Alpha* and *beta* oscillations, Dynamic stimuli, Goal-directed behavior, Magnetoencephalography (MEG), Spatial attention, Attentive tracking, Action observation

## Abstract

Allocation of attention during goal-directed behavior entails simultaneous processing of relevant and attenuation of irrelevant information. How the brain delegates such processes when confronted with dynamic (biological motion) stimuli and harnesses relevant sensory information for sculpting prospective responses remains unclear. We analyzed neuromagnetic signals that were recorded while participants attentively tracked an actor's pointing movement that ended at the location where subsequently the response-cue indicated the required response. We found the observers' spatial allocation of attention to be dynamically reflected in lateralized parieto-occipital *alpha* (8–12 Hz) activity and to have a lasting influence on motor preparation. Specifically, *beta* (16–25 Hz) power modulation reflected observers' tendency to selectively prepare for a spatially compatible response even before knowing the required one. We discuss the observed frequency-specific and temporally evolving neural activity within a framework of integrated visuomotor processing and point towards possible implications about the mechanisms involved in action observation.

## Highlights

• Observing dynamic actions modulates on-going *alpha* and *beta* neural activity.
• *Alpha* modulations reflect dynamic changes in the allocation of spatial attention.
• *Beta* modulations relate to evolving, stimulus location-based response bias.
• *Alpha* and *beta* activity jointly contribute to sensorimotor integration for action.

## Introduction

When interacting with our environment, we face the problem of selecting among a host of action possibilities (Cisek and Kalaska, 2010) that are afforded by different agents and/or objects that attract our attention to varying degrees (e.g., Gibson, 1966; Grèzes et al., 2003). An effective way to resolve this challenge is through directed visuo-spatial attention, which has been repeatedly shown to improve processing of information falling within the locus of attention while reducing the interference from competing sensory information occurring elsewhere within the visual field (Desimone, 1998; Desimone and Duncan, 1995; Posner et al., 1980). Attention studies have frequently examined static stimulation conditions, however, and in part constrained by neuroimaging methods with limited temporal resolution (Culham et al., 1998), much less is known about the brain mechanisms supporting dynamic attention processes (e.g., as involved in attentional tracking of moving objects in space). This is particularly so in everyday contexts such as action observation, an activity known to engage both attention (Belopolsky et al., 2008) and motor systems (Koelewijn et al., 2008).

Insights into the role of brain oscillations involved in spatial attention processes have particularly been gained through human neurophysiological studies employing the endogenous pre-cuing paradigm (Posner et al., 1980). In this paradigm, a cue informs about the likely location of a laterally presented target stimulus. After a delay period (cue–target interval of ~ 1.0 to 2.5 s) the target stimulus then demands participants to make a perceptual detection (Thut et al., 2006) or discrimination response (Siegel et al., 2008). Cumulative research evidence has shown that lateralized parieto-occipital *alpha* (8–12 Hz) oscillations are strongly associated with the deployment of spatial attention (Capila et al., 2012; Thut et al., 2006) as well as modulated by spatial certainty (Gould et al., 2011). Specifically, the decrease of *alpha* oscillation amplitude in the hemisphere contralateral to the attended visual hemifield has been related to enhanced processing of information at the attended location, whereas the increase (or constant) *alpha* oscillation amplitude in the hemisphere ipsilateral to the attended visual hemifield has been related to the suppression of processing of information at the unattended location (Rihs et al., 2009).

In addition to the observation that *alpha* neural activity is associated with the functional inhibition of task-irrelevant brain areas (Händel et al., 2010; Jensen and Mazaheri, 2010; Kelly et al., 2006), other studies have also demonstrated that lateralized *alpha* amplitude is (inversely) related to perceptual performance (Hanslmayr et al., 2007; van Dijk et al., 2008; Wyart and Tallon-Baudry, 2009). Thus, improvement of both perceptual and motor performance correlates with the suppression of parieto-occipital *alpha* oscillations (Foxe and Snyder, 2011). Since occipital *alpha* oscillations have been linked to excitability changes of visual areas (Romei et al., 2008a, 2008b, 2010) modulations of perceptual performance are not surprising. However, response times depend on the activation state of motor areas that is typically reflected in *beta* (16–35 Hz) oscillations (Engel and Fries, 2010). In fact, *beta* oscillations in cortical motor areas are known to have a causal effect on movement duration, force-generation, and inhibition (Joundi et al., 2012; Pogosyan et al., 2009) and are modulated during action observation (Kilner et al., 2009; Press et al., 2011). Yet, the specific contributions of parieto-occipital *alpha* and motor *beta* oscillations to perceptually (e.g., spatially) invigorated action preparation remain unclear. Indeed, we know relatively little about how *alpha* oscillations (i) mediate the expectancy-related attention process, and (ii) influence the on-going neural dynamics involved in shaping our prospective actions when spatial “cue” information is dynamically provided rather than statically some time before target onset. However, the former scenario mimics an everyday situation (e.g., driving through a busy crossroad where the movement of other vehicles and pedestrians require simultaneous monitoring) wherein we are required to pay attention (overtly or covertly) to dynamically moving object(s) of interest before deciding which action will be taken shortly.

**Fig. 1:**
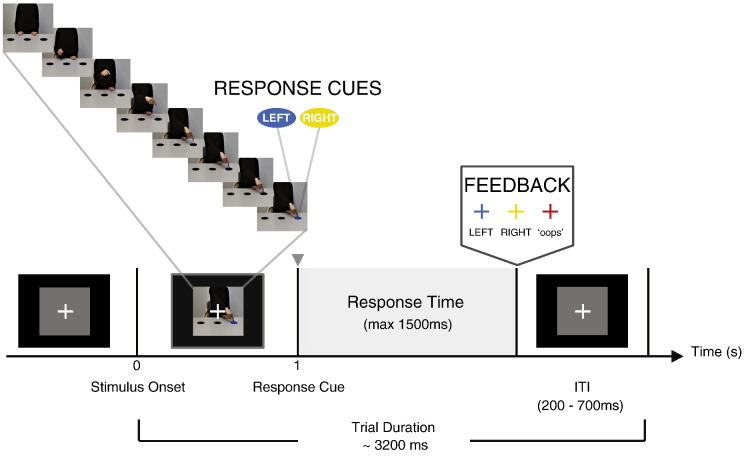
The sequence of events in an experimental trial. A gray background screen of equal size as the movie frames (13° × 13° visual angle) was presented for a randomized inter-trial interval (ITI) of 200 to 700 ms. The movie (60 fps) started with the presentation of the first frame at *t* = 0 ms. The last movie frame and the response cue appeared at *t* = 1000 ms, and remained on screen showing the actor with the pointing hand in its end position and the other hand in a resting position. A target color change from black to blue (yellow) necessitated a left (right) index response within 1500 ms post response cue onset. In trials requiring no response, the randomized ITI followed 1500 ms after the end of the pointing movement. Throughout the experimental session a white fixation cross was centered in the middle of the stimuli, and it was also present during the randomized inter-trial intervals when the movie still-frames were reset to a gray background. Participants were provided with response feedback during the ITI in the form of a color change in the fixation cross that corresponded to their actual response (i.e. blue for ‘left’, yellow for ‘right’, and red for a ‘wrong’ response, such as an incorrect or delayed response).

The present study investigates how attention is allocated in the presence of dynamic biological motion stimuli (i.e., the arm movement of an actor) and how on-going anticipatory motor activation is influenced. More specifically, we used magnetoencephalography (MEG) to monitor participants' *alpha* and *beta* oscillations while they observed an actor making pointing movements towards a lateral target (Fig. 1). At the end of the pointing movement the color of the target changed and cued the participant to perform a right or left hand response. Importantly, the actor's pointing movements were either targeted towards an endpoint in the same hemifield (straight movement) or one located in the opposite hemifield (crossed movement), thereby validly indicating the position of the response cue but not that of the to-be-executed response. The task as a whole requires participants i) to detect/discriminate the pointing hand in the spatial periphery, ii) to attentively track the changing spatial location of the pointing hand, and iii) to discriminate the response cue after the pointing movement reached its endpoint location. This modified ‘Simon-task’ (Simon, 1969) provides a useful experimental paradigm for a number of reasons. First, it fulfills the attention processing criteria as employed in previous visuo-spatial attention research. Second, it grounds the task within an ecological framework as we deal with both static and dynamic stimuli in our daily interactions with others. Third, and importantly, our modified task version allows us to temporally segregate actual motor from sensory processing and to focus specifically on the attention-related processes prior to any actual movement onset. Furthermore, presenting straight and crossed arm movements allows us to test whether covert motor activation induced during action observation (Koelewijn et al., 2008) relates to the moving limb of the actor (effector mirroring) or rather to the spatial position of the arm (position mirroring). Whereas behavioral evidence regarding this issue is mixed (Belopolsky et al., 2008; Bertenthal et al., 2006), to our knowledge, there is only one functional neuroimaging (MEG) study that addressed this issue (Kilner et al., 2009), finding evidence against the effector mirroring view.

Our study specifically focuses on the sensory and cognitive processes leading up to motor response anticipation. We hypothesize that the active allocation of attention to incoming visuo-spatial information serves to dynamically update predictions about the behaviorally relevant movement endpoint (which could be located either in observers' left or right visual hemifield). Consequently, the emerging *alpha* lateralization in parieto-occipital brain regions should sensitively index the associated shift in spatial attention. It is also known that *beta* oscillations in cortical motor regions are related to the temporal prediction of sensorimotor events (Fujioka et al., 2012; Saleh et al., 2010) and the state of motor preparation as a function of response certainty (Tzagarakis et al., 2010). Therefore, we further hypothesize to observe dynamic changes in *beta* lateralization reflecting anticipatory processes, e.g., response bias, and/or influences of sensorimotor information that can be expected to change during the attentive observation of the actor's pointing movement. Crucially, source-space analysis will allow us to gain first insights into the temporal interplay of *alpha* and *beta* oscillatory activity in brain areas concerned with sensory, attentional, and motor processing. Our time-resolved MEG sensor- and source-space analyses corroborated these hypotheses, demonstrating the dynamic updating of spatial attention by incoming sensory evidence together with priming of parieto-premotor areas. These results further inform current views regarding some of the key brain processes involved in the action observation network (e.g., Kilner, 2011; Thompson and Parasuraman, 2012).

## Materials and methods

### Participants

Twelve healthy right-handed (Oldfield, 1971) paid volunteers (6 females, mean age 24.4 years; SEM ± 1.8) with no history of neurological illness participated in the study after providing informed consent. The study was approved by the College of Science and Engineering Ethics Committee (University of Glasgow).

### Stimuli and task

Participants were shown 1 s-movies of an actor, seated forward-facing, making a pointing action to one of two lateral targets located in front of both the actor and participant (Fig. 1). The actor's body and arms/hands were visible but not the face. The movies began (*t* = 0 ms) with the actor resting both hands palms down on the table, prior to making a pointing action with either the left or right hand. The movement terminated with the actor's moving index finger ending on one of the lateral targets. Critically, on the last frame of the movie (*t* = 1000 ms), the target that the actor pointed to either changed in color from black to blue or yellow, or it remained black. This target color change provided participants the relevant response cue. A target color change from black to blue (yellow) required a left (right) index finger response, while the response should be withheld in trials with no target color change. The stimuli were presented with an inter-trial-interval of 200 to 700 ms post response (or after 1500 ms in no-response trials) using the Psychophysics Toolbox (v3.0.8) (Brainard, 1997; Pelli, 1997) within MATLAB ® (MathWorks™, MA, USA). Twelve different *Experimental Conditions*, from the factorial combination of (i) the actor's moving hand (left vs. right), (ii) the target location corresponding to the movement endpoint (left vs. right, relative to the observer) and (iii) the required response (left, right or none), were each presented 10 times in randomized order in each of the eight experimental blocks. Each block consisted of a randomized sequence of 120 trials.

### Neuroimaging acquisition

Participants were tested sitting upright within an electromagnetically shielded room. MEG data were acquired using a 248-channel magnetometer system (Magnes 3600 WH; 4D Neuroimaging, San Diego, USA). Head position stability was assessed via five head-position indicator coils attached relative to the (left, right preauricular and nasion) fiducials which were co-digitized with head-shape (FASTRAK®, Polhemus Inc., VT, USA) for subsequent co-registration with individual MRI (1 mm^3^ T1-weighted; 3D MPRAGE). The MEG, index finger responses (LUMItouch™, Photon Control Inc., BC, Canada) and eye-tracker (EyeLink 1000; SR Research Ltd., Ontario, Canada) signals were sampled synchronously at 1017.25 Hz.

### Behavioral analysis

**Fig. 2:**
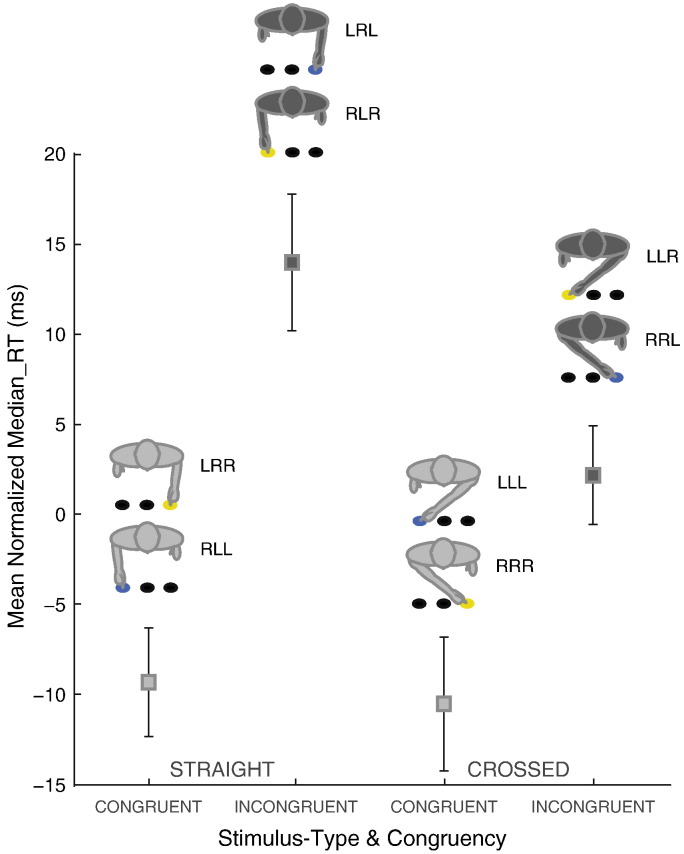
Normalized median response time (RT) as a function of *Stimulus-Type* (straight vs. crossed) and *Response*–*Congruency* (congruent vs. incongruent). Error bars depict the standard error of normalized mean RT. *Stimulus-Type* conditions (denoted as LR ~; RL ~; LL ~; RR ~), in which participants viewed straight (i.e., LR ~ or RL ~) or crossed (i.e., LL ~ or RR ~) pointing hand movements are factorial combination of (*A*) the actor's moving hand and (*T*) the endpoint target location of the hand movement relative to the observer, while (*R*) the cued response (denoted by ‘~’) was irrelevant. Similarly, *Response*–*Congruency* conditions (denoted as ~ LR; ~ RL; ~ LL; ~ RR; ~ signifies the irrelevance of the actor's moving hand), in which participants make a response that is spatially congruent (i.e., ~ LL or ~ RR) with the endpoint target location of the actor's movement or not (i.e., ~ RL or ~ LR). Median RTs ranged from 300 to 610 ms (mean ± SEM = 451 ± 10 ms) across subjects and response-required *Experimental Conditions*.

Individual median response times (RT) were determined for the eight response-required *Experimental Conditions* (Fig. 2). We assessed the effects of (*A*) the *Actor's Moving Hand* (left vs. right), (*T*) the *Endpoint Target Location* on which the actor's pointing movement ended (left vs. right, relative to the participant's perspective), and (*R*) the *Cued Response* (left vs. right) on participants’ response with repeated measures analysis of variance (PASW Statistics 18, SPSS Inc., IBM, IL, USA).

### MEG data processing

All data processing, time–frequency and statistical analyses were performed using Fieldtrip (Oostenveld et al., 2011) within MATLAB®. During MEG acquisition, there were a few noisy channels that were invariant across subjects and some that were subject-specific. To standardize the whole signal pre-processing and to facilitate the subsequent source analysis, a common set of MEG sensors (N = 26, visually identified, and located primarily in the frontal sensors) with large signal variance was removed from the MEG data set. Next, for sensor-level analysis, we performed nearest-neighbor interpolation of the removed noisy channels using the Fieldtrip function *ft_channelrepair*. In subsequent sensor-level analyses, this allowed us the use of the same set of 248 channels across all subjects. Raw MEG signals were epoched from − 1000 to + 3000 ms relative to stimulus onset (0 ms), with linear trends removed. Eye-blinks and movement artifacts were rejected through trial-by-trial visual inspection. The remaining epochs were ‘de-noised’ relative to reference MEG signals prior to Independent Component Analysis to isolate and reject the cardiac component from the MEG signal.

Artifact-free neuromagnetic time series (mean (SEM) = 541.25 ± 17.24 trials) corresponding to correct trials were transformed to planar gradient signals (Bastiaansen and Knosche, 2000) that entered subsequent time–frequency analyses. For each of the eight response-required *Experimental Conditions* (Fig. 2), time–frequency time-series were computed from − 1000 ms to 3000 ms using a Hanning-tapered 500 ms temporal window and a 20 ms time resolution. These time-series were expressed as a relative change to baseline.

### Sensor-level analysis

**Fig. S1:**
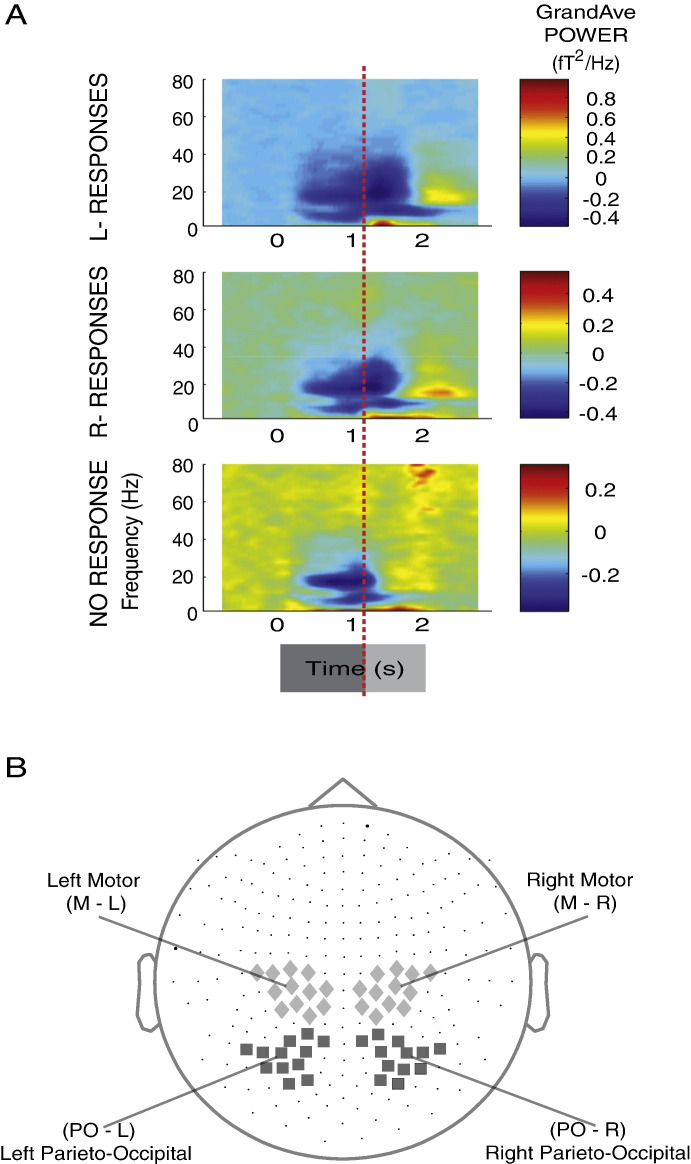
Selection of sensor clusters. (A) Grand-averaged time–frequency power modulations for trials requiring left, right, or no responses. The red vertical line separates the perception-related temporal range of interest (darker gray temporal bar; 0 to 1250 ms) and the movement-related temporal range of interest (lighter gray temporal bar; 1250 to 1750 ms relative to stimulus onset). (B) Selected clusters of sensors over left and right hemispheric motor and parieto-occipital areas. Perception- and movement-related MEG sensor clusters are indicated by light and dark gray shading, respectively.

Four groups of MEG sensors were defined that covered left/right parieto-occipital and left/right motor areas (see Supplementary Methods section; Inline Supplementary Fig. S1). Subsequent analyses were performed using the relative power change spectra (${\bar{\Delta P}}_{f_{\left(t\right)}}$) obtained from these sensor subsets and their lateralized signals (i.e., subtraction of the relative change in power spectra for right-hemispheric sensors from that corresponding to the left-hemispheric sensors). Hemisphere-specific and lateralized neuromagnetic modulations in *alpha* (8–12 Hz) and *beta* (16–25 Hz) frequency (*f*) bands were derived for the required conditions (*c*). The effect of (*A*), (*T*), and (*R*) on *alpha* and *beta* neural activity was examined using time-resolved regression analysis. We used condition-specific averaged motor or parieto-occipital spectra from the left (${\bar{\Delta P}}_{L\;f_{\left(t\right)}}^{c,s}$) or the right (${\bar{\Delta P}}_{R\;f_{\left(t\right)}}^{c,s}$) hemisphere, or the lateralization (${\bar{\Delta P}}_{\mathrm{Lat}\;f_{\left(t\right)}}^{c,s}$) of each participant (*s* = 1 to 12) as dependent variables and specified independent variables as pseudo dummy values, − 1 or 1 for left or right, respectively, with reference to the *Actor's Moving Hand* (*A*), *Endpoint Target Location* relative to the observer's perspective (*T*), and *Cued Response* (*R*) as defined in *Behavioral analysis*. This is generalized as:

(1) $${\bar{\Delta P}}_{\mathrm{hem}\;f_{\left(t\right)}}^{\;c,s}=b_{0}+b_{1}A_{s}^{c}+b_{2}T_{s}^{c}+b_{3}R_{s}^{c}+\varepsilon$$

**Fig. 3:**
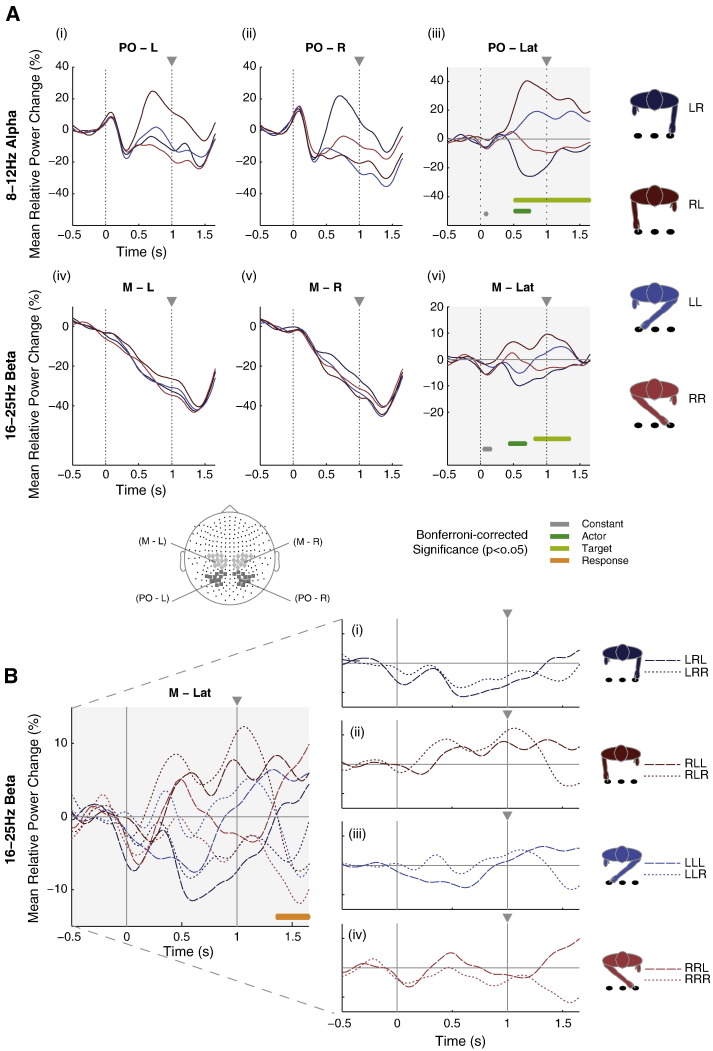
Relative time–frequency power change modulation for *alpha* and *beta* oscillations. (A) *Stimulus-related spectral modulation*: temporally evolving parieto-occipital (PO) *alpha* power (A_(i),(ii),(iii)_) modulations during different pointing movements (*Stimulus-Types* color-coded) in the left (PO-L; A_(i)_), right (PO-R; A_(ii)_), and lateralized (i.e., left minus right; PO-Lat; A_(iii)_) hemispheric *alpha* power time series as well as the left (M-L; A_(iv)_), right (M-R; A_(v)_), and lateralized (i.e., left minus right; M-Lat, A_(vi)_) hemispheric motor *beta* power time series. Time points for which the regression analysis revealed frequency-specific lateralized relative power change in both motor and PO time series to be significantly associated with actor's hand, target location, and response hand are depicted by the colored horizontal lines below the power modulation plots (A_(iii),(vi)_). (B) *Response-related spectral modulation*: mean lateralized relative *beta* power change time series for all *Experimental Conditions*. Thick vs. fine dashed lines represent lateralized power modulation in scenarios where a left vs. a right hand response is required, respectively. The orange horizontal line below the power modulation plot indicates the time points for which the frequency-specific lateralized relative power change was significantly associated with the response hand. Condition specific modulations are illustrated in the subplots B_(i),(ii),(iii),(iv)_.

${\bar{\Delta P}}_{\mathrm{hem}\;f_{\left(t\right)}}^{c,s}$ denotes the motor or parieto-occipital spectra from the left or right hemisphere, or the lateralized spectral time series of each participant (as defined above), *b*_0,1,2…*N*_ are the regression coefficients and *ε* the residual error for each sample time point of interest (*t*) within − 500 to + 1650 ms relative to stimulus onset. The regression coefficients corresponding to the parameters of interest (*A*, *T*, *R*) were assessed using Bonferroni-corrected (p < 0.0004) *t*-tests (Manly, 2007). The color-coded bars depicted underneath the grand-averaged PO- and M-Lateralized spectral time series (Fig. 3A_(iii),(vi)_) indicate the significant time points. For simplicity, Fig. 3A shows the grand-average spectral time series for *Stimulus-type* conditions (LR ~; RL ~; LL ~; RR ~; Fig. 2), in which participants viewed straight or crossed pointing hand movements. These four conditions resulted from the factorial combination of (*A*) the *Actor's Moving Hand* and (*T*) the *Endpoint Target Location* of the hand movement relative to the observer, while (*R*) the *Cued Response* (denoted by ‘~’) was irrelevant.

Inline Supplementary Figure S1

### Lateralized *beta* power modulations and RT

Based on the significant interactions between endpoint location and required responses (see Supplementary Results section and Inline Supplementary Table S1) lateralized *beta* power time series for the eight *Experimental Conditions* were grouped by *Response*–*Congruency* (denoted as ~ LR; ~ RL; ~ LL; ~ RR; ~ signifies the irrelevance of the actor's moving hand; Fig. 2). We accounted for laterality effects of response-related neural activity by multiplying experimental conditions requiring left or right responses with − 1 or 1, respectively. These power spectra were normalized by each subject's maximum spectral power across all *Experimental Conditions* during stimulus presentation (0 to 1000 ms). For each sampling time point (20 ms resolution) during the experimental trial (− 500 to 2000 ms), the relation between normalized power spectra for each *Response*–*Congruency* condition and the corresponding normalized median RT was assessed by Pearson correlation.

Inline Supplementary Table S1

**Table S1:** Summary of analysis of variance analyses (ANOVA) assessing effects of salient factors on median response times (RT). 3-way ANOVA was performed with (i) *Actor's Moving Hand*, (ii) *Cued Response Hand*, and (iii) *Endpoint Target Location* (abbreviated as *A*, *R*, *T*, respectively) as salient factors. 2-way ANOVA was performed with combined *Experimental Conditions*: (viii) straight or crossed *Stimulus–Type* and (ix) *Response–Congruency* (abbreviated as *S-type* and *R*–*congr.*, respectively) as salient factors. Statistical significance is indicated as follows: n.s. (non-significant); * (p < 0.05); ** (p < 0.005). Refer to text in the Results section and the Supplementary Results section for further details.

	Main Effects	Abbrev.	Contrasts	Median RT (ms) mean ± SEM	Fstats F(1,11)	p	Sigf.
i	*Actor’s Moving Hand*	*A*	Left	450 ± 18	0*.*797	0*.*391	n.s.
Right	453 ± 18
ii	*Cued Response* *Hand*	*R*	Left	454 ± 19	0*.*476	0*.*505	n.s.
Right	448 ± 18
iii	*Endpoint Target* *Location*	*T*	Left	453 ± 18	18*.*545		**
Right	449 ± 18
iv	Interaction	*A* × *R*			4.898	0.049	*
v	Interaction	*T* × *A*			9.602	0.010	*
vi	Interaction	*T* × *R*			10.013	0.009	**
vii	Interaction	*A* × *T* × *R*			5.073	0.046	*
viii	*Stimulus-type*	*S*-*type*	Straight	455 ± 18	9*.*602	0.010	*
Crossed	448 ± 18
ix	*Response*–*Congruency*	*R*–*congr*.	Congruent	442 ± 19	10.013	0.009	**
Incongruent	460 ± 18
x	Interaction		*S-type* × *R*–*congr.*		4*.*898	0*.*049	*

Inline Supplementary Table S1

### Source-level analysis

To further investigate the underlying neural sources contributing to the observed effects in the sensor-level analysis, we performed lateralized (left vs. right hemisphere) contrast analyses at source level within subjects prior to group comparisons and assessed the regional source maxima to determine brain regions (ROIs) significantly related to *Stimulus-Type* and *Response*–*Congruency*. Noisy or interpolated channels were excluded, that is, all source-level analyses were conducted using the 222 good channels. Time–frequency source signals (600 to 1100 ms; baseline − 500 to 0 ms) were derived using DICS (Gross et al., 2001) with individual's MRI (6 mm volume grid, normalized to the MNI space) and experimental session-specific sensor location to compute forward modeling lead fields. Spatial filters for localizing *alpha* and *beta* activities were derived with a linear constraint allowing maximal gain of source signal power while maximally suppressing that of all other sources and minimizing overall output signal variance (Van Veen et al., 1997). Individual baseline-contrasted *stimulus-* and *response-related* statistical source maps were subsequently analyzed by performing group-level contrast analyses to investigate significant sources pertaining to 1) *Stimulus-Type* (i.e., straight (LR ~ vs. RL ~) vs. crossed (LL ~ vs. RR ~) pointing movements), and 2) *Response*–*Congruency* (i.e., congruent (~ LL vs. ~ RR) vs. incongruent (~ LR vs. ~ RL) responses). Results were bootstrap-resampled (N = 500) to estimate confidence intervals and FDR corrected (*α* = 0.05).

**Fig. S2:**
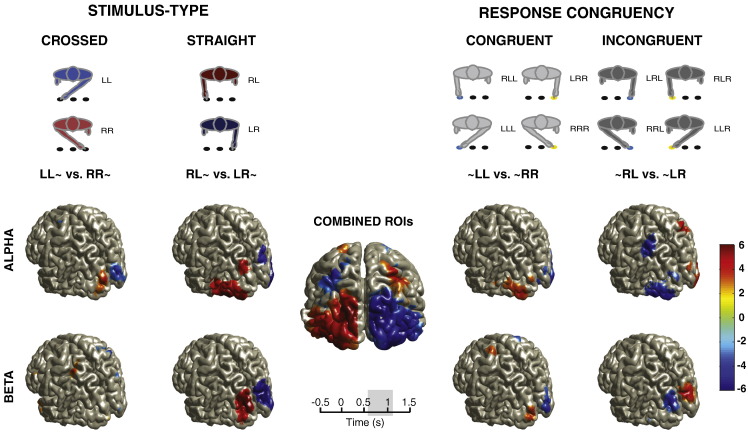
Significant brain sources related to *Stimulus–Type* and *Response–Congruency* in *alpha* and *beta* frequencies between 600 and 1100 ms after stimulus onset. Relative changes in time–frequency power modulations of derived brain sources were compared between hemispheres to validate lateralization effects. The color bar depicts FDR corrected *t*-statistic values corresponding to the significance of source contrast comparisons projected onto the brain surface. (Note: Depending on the conditional contrasts, e.g., *Stimulus–Type* or *Response–Congruency*, the “L minus R” contrast comparisons were made with respect to either the endpoint Target location or the Response hand, respectively. As such, the positive (red) and negative (blue) contrast *t*-statistics may appear flipped in some cases (i.e., incongruent response comparison; ~ RL vs. ~ LR) on the projected brain surface.). The central brain surface plot depicts the combined significant brain sources and their regional absolute maxima were selected as regions of interest (ROIs; see “*Source-level analysis*” in Materials and methods section and Supplementary Table S1 for further details) in subsequent source-space analysis relating to response times. The brain surface plots are rendered by projecting sources maximally activated within 5 mm of brain volume, with projection threshold = 55% of the maximal *t*-statistic value.

Significant source localizations were integrated within the same brain grid space (Fig. 5; see Inline Supplementary Fig. S2). We derived from the local maximum of these significant sources a set of bilateral ROIs known to be involved in sensory-motor integration (e.g., Donner et al., 2009; Ledberg et al., 2007) and importantly, functionally related to the overall task by deriving the regional cortical maxima (based on source comparison *t*-test statistics) with at least four significant connected surrounding voxels, and that their combined absolute mean of statistics value *t* ≥ 2.6. These maxima and their corresponding contralateral location contributed to the set of bilateral ROIs (N = 10; see Inline Supplementary Fig. S2 and Table S2).

Inline Supplementary Figure S2

Inline Supplementary Table S2

**Table S2:** Statistically-determined task-relevant ROIs. Bilateral ROIs were derived from the combined statistically significant *Stimulus–Type* and *Response–Congruency* contrasts' regional maxima. MNI coordinates were used to find corresponding anatomical labels within the Fieldtrip toolbox (using the function *ft_prepare_atlas* which calls and accesses the AFNI brik file that is available from http://afni.nimh.nih.gov/afni/doc/misc/ttatlas_tlrc). For further details see “*Source-level analysis*” in Materials and methods section.

ROI#	MNI			Cerebral hemisphere	Lobe	Landmark	Brodmann area (BA)	Text label	Hemispheric regional max voxel density	Mean (FDR stats)
X	Y	Z
1	18	− 18	64	Right	Frontal	Precentral gyrus	BA 6	PMd	4	2*.*79
2	− 18	− 18	64	Left	Frontal	Precentral gyrus	BA 6
3	54	− 64	8	Right	Temporal	Middle temporal gyrus	BA 37	pMTG	6	4*.*35
BA 39
4	− 54	− 64	8	Left	Temporal	Middle temporal gyrus	BA 37
BA 39
5	12	− 84	22	Right	Occipital	Cuneus	BA 18	BA 18	18	3*.*93
6	− 12	− 84	22	Left	Occipital	Cuneus	BA 18
7	18	− 84	40	Right	Parietal	Precuneus	BA 19	BA 19	21	4*.*56
8	− 18	− 84	40	Left	Parietal	Precuneus	BA 19
9	36	− 48	58	Right	Parietal	Superior parietal lobule	BA 7	PPC	6	2*.*79
Inferior parietal lobule	BA 40
10	− 36	− 48	58	Left	Parietal	Superior parietal lobule	BA 7
Inferior parietal lobule	BA 40

Inline Supplementary Table S2

### Lateralized ROI source time–frequency power and RT

We derived lateralized baseline-corrected ROI power time series (Lat_ROI) in *alpha* and *beta* frequency bands. Power was calculated as square of the absolute complex signals from DICS with fixed dipole orientations and baseline-corrected as relative change. For every 100 ms moving average (50 ms resolution) from stimulus onset (0 ms) until after the response (1650 ms), we tested the correlation between source Lat_ROI and RT. These moving average correlations were categorized according to their statistical significance (uncorrected) into five p-value threshold bins (n.s.; p < 0.05; p < 0.005; p < 0.0001; p < 0.00001; Fig. 5).

## Results

### Dynamic viewing of actions has lingering spatial effects on behavior

When sequentially attending to (*A*) *Actor's Moving Hand*, (*T*) *Endpoint Target Location*, and (*R*) *Cued Response* participants' median RT was significantly driven by the interaction between endpoint target location and actor's moving hand (F_(1,11)_ = 9.602, p = 0.010), and the interaction between endpoint target location and cued response (F_(1,11)_ = 10.013, p = 0.009). The three-way interaction among all three factors reached significance (F_(1,11)_ = 5.073, p = 0.046; see Inline Supplementary Results section and Inline Supplementary Table S1, i–vii). Given the non-significant RT main effects relating to the actor's moving hand and cued response (Fs < 1, p > 0.39), we simplified our analyses by combining the eight response-required *Experimental-Conditions* according to *Stimulus-type* (i.e., straight vs. crossed movement) and *Response*–*Congruency* (i.e., congruent vs. incongruent response). This yielded four *Stimulus*–*response categories*: 1) straight-response congruent; 2) straight-response incongruent; 3) crossed-response congruent; 4) crossed-response incongruent (Fig. 2). Participants made faster congruent compared to incongruent responses (mean ± SEM = 442 ± 19 ms vs. 460 ± 18 ms; F_(1,11)_ = 10.013, p = 0.009) and faster responses when observing the actor performing crossed compared to straight pointing movements (mean ± SEM = 448 ± 18 ms vs. 455 ± 18 ms; F_(1,11)_ = 9.602, p = 0.010). The interaction between *Stimulus-type* and *Response*–*Congruency* reached significance (F_(1,11)_ = 4.898, p = 0.049; Inline Supplementary Table S1,viii–x), reflecting the three-way interaction.

### Attentive tracking of pointing movements is reflected in *alpha* and *beta* neural modulations

During participants' viewing of the Actor's different pointing movements (*Stimulus-Types*; Fig. 3A), parieto-occipital (PO) and motor (M) sensor-based (Fig. 3) neural power showed distinctive modulations in *alpha* and *beta* oscillations. Sharp and brief PO *alpha* oscillatory power decrease manifested itself early in both hemispheres, falling ~ 20% below baseline ~ 275 ms following stimulus onset (Fig. 3A_(i);(ii)_). Thereafter, modulations related to *Stimulus-Type* were seen to deviate from the point where the early *alpha* oscillatory power reduction was maximal (~ 300 ms). Attentive tracking of straight pointing movements was associated with the strongest rebound of *alpha* oscillatory power (~ 40% relative power increase from initial minima, peaking at ~ 700 ms) in either hemisphere contralateral to the endpoint target location. The *Stimulus-Type* related modulations were distinctly separated prior to response cue onset (1000 ms) in each hemisphere. This is clearly seen in the lateralized spectral time series (Fig. 3A_(iii)_). In particular, lateralized *alpha* activity reflected coding of the location of the target to which the actor's hand is moving ~ 500 ms after stimulus onset. Straight pointing movements, where the actor's hand stays within the observer's left or right visual field, elicited enhanced ipsilateral *alpha* power modulation with corresponding contralateral suppression. For crossed pointing movements, *alpha* modulation followed a similar but weaker trend.

Distinctive motor *beta* power changes decreased (to ~ 50% below baseline) from stimulus onset until the period of response onset (at ~ 1450 ms) before rebounding for all *Stimulus-Type* in both hemispheres (Fig. 3A_(iv);(v)_). From ~ 400 ms after stimulus onset, lateralized motor *beta* modulations (Fig. 3A_(vi)_) appeared to code the spatial position of the actor's hand as reflected by lower *beta* amplitude in contralateral compared to ipsilateral motor areas. This is most obvious when the actor made crossed pointing movements, i.e., when the actor's left hand moved from the participants' right to the left hemi-field (experimental condition LL). In this case, the participants' initially negative motor *beta* lateralization (L < R) gradually shifted to become positive (L > R).

### Evolving ‘saliency’ of observed stimuli dynamically modulates neuromagnetic signals

Sequential unfolding of significant associations (p < 0.05, Bonferroni corrected) between lateralized spectral power changes (relative to baseline) and specific ‘salient’ features of *Experimental Conditions* can be observed between stimulus (0 ms) and response cue (1000 ms) onsets, and prior to the mean time of response onset (~ 1450 ms; Fig. 3A_(iii),(vi)_; olive and lime green significance lines). Specifically, the regression analysis revealed that lateralized motor *beta* modulation (Fig. 3A_(vi)_) was significantly associated with actor's moving hand, beginning at 427 ms post stimulus onset. Thereafter, lateralized PO *alpha* modulation (Fig. 3A_(iii)_) that was significantly related to both the actor's moving hand and the endpoint target location emerged at 508 ms post stimulus onset. Significant target-related associations were next observed in lateralized motor *beta* at 810 ms (Fig. 3A_(vi)_), that is 190 ms before response cue onset. Subsequently, we observed a significant association between lateralized motor *beta* and the required hand response preceding its execution at 1355 ms (Fig. 3B; orange significance line).

The regression analysis revealed that past the midpoint of the pointing movement, observers' lateralized PO *alpha* modulation was briefly, but significantly, related to the actor's moving hand before manifesting a significant relation to the target location (Fig. 3A_(iii)_). Lateralized motor *beta* power also revealed significant modulations that transited from reflecting the position of the actor's moving hand to that of the target location (Fig. 3A_(vi)_). Such a *beta* modulation is clearly observable for the (crossed) right hand pointing movement to the right target (Fig. 3B_(iv)_, experimental condition RR). In this condition, prior to response cue onset, mean lateralized motor *beta* suppression indexed the engagement of motor areas for a right hand response, probably based on the inference of the actor's movement endpoint. When the response cue indicated a congruent right hand response, this decrease in *beta* oscillatory power was further enhanced. However, when an incongruent left hand response was cued, the lateralized motor *beta* power modulation showed a ‘reversal’, indexing the engagement of motor areas in the other hemisphere for the required left hand response. Such lateralized spectral modulation was similarly observed for the other *Stimulus-Type* and corresponding *Response*–*Congruence* conditions (Fig. 3B_(i),(ii),(iii)_).

**Fig. 4:**
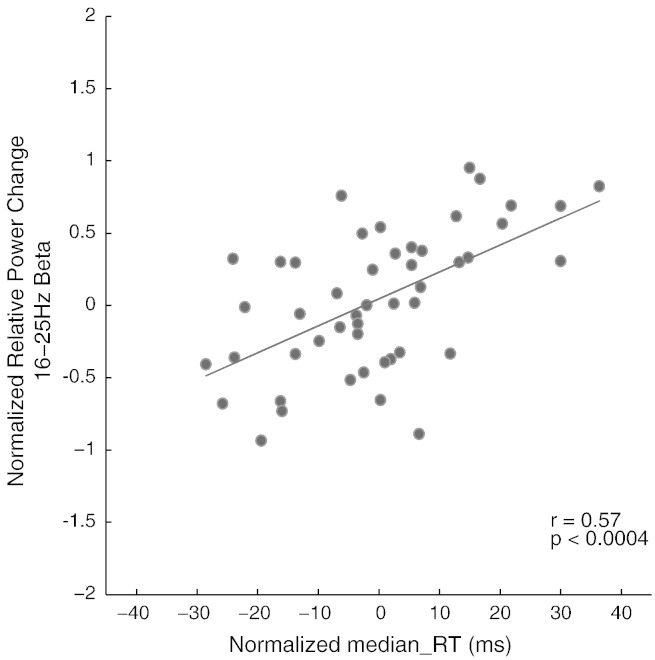
Correlation between normalized lateralized motor *beta* power and median response time (RT) across subjects at 100 ms period prior to response cue onset (at *t* = 1000 ms).

Importantly, the observation of a stronger decrease in *beta* oscillatory power in the hemisphere contralateral (cf. ipsilateral) to the endpoint target location prior to response cue onset suggests that the lateralized *beta* modulation might reflect participants' bias towards a congruent response, based on their inference of the endpoint target location (or the actor's movement goal). Further investigation of the association between lateralized motor *beta* power and RT confirmed this hypothetical response bias, yielding a significant correlation at 100 ms before response cue onset (r = 0.57, p < 0.0004, Bonferroni corrected; Fig. 4), i.e., even before participants knew with which hand to respond.

### Dynamic interplay of *alpha* and *beta* processes across ROIs as participants gear up for action

Regional maxima, i.e., occipital visual (BA 18, BA 19), posterior middle temporal gyrus (pMTG; BA 37, BA 39), parietal (PPC; BA 40, BA 7), and dorsal premotor (PMd; BA 6) areas, were derived using frequency-specific source localizations (see Supplementary Results section; Inline Supplementary Fig. S2) using the frequency-specific contrast comparisons (i.e., straight vs. crossed *Stimulus-type*; congruent vs. incongruent *Response*–*Congruency*). The bilateral coordinates of these maxima were selected as regions of interest (ROIs) for further investigation (see Inline Supplementary Table S2).

**Fig. 5:**
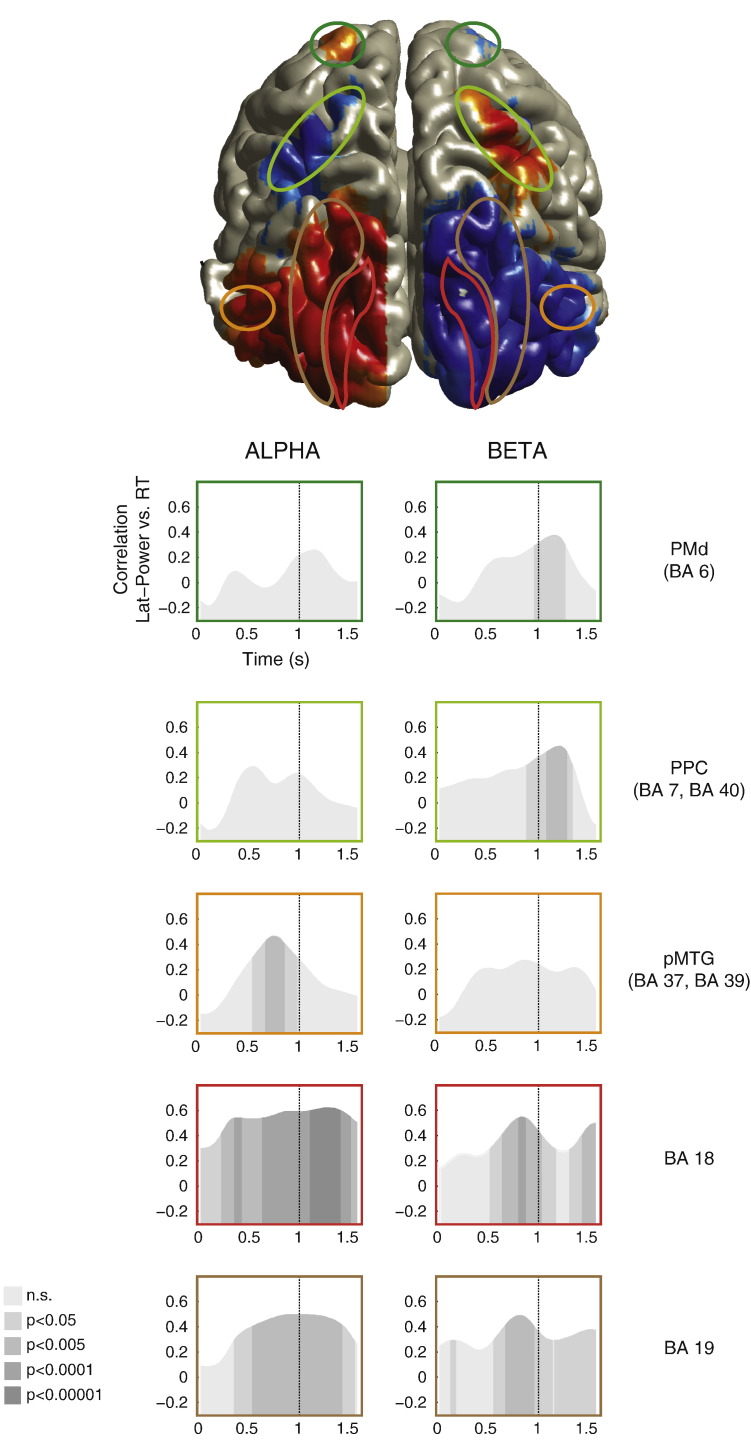
(Above) The central brain surface plot depicts the combined significant brain sources and their regional absolute maxima were selected as regions of interest (ROIs; see “Contrast comparison source localizations” in Supplementary methods, Supplementary Fig. S2, and Supplementary Table S1 for further details). (Below) Time evolving correlation between mean lateralized source ROI time–frequency power and behavior RT in 100 ms steps from 0 to 1500 ms relative to stimulus onset in parietal (PPC) and premotor (PMd) areas as well as visual areas (BA 18, BA 19, and pMTG) separately for *alpha* and *beta* frequencies (left vs. right column). The gray-scale shading indicates the level of the (uncorrected) correlation significance (n.s.; p < 0.05; p < 0.005; p < 0.0001; p < 0.00001).

The moving average correlation revealed an evolving strength of association between the frequency-specific lateralized power modulations from these paired ROIs and RT (Fig. 5; Inline Supplementary Table S3A). Predominantly, *alpha* oscillatory processes within perceptual brain regions (BA18, BA19) manifested early (at 50 and 150 ms, respectively) correlations with RT. Despite increases in correlation between RT and *alpha* power within PMd and PPC areas over time, these were non-significant. The strength and significance of correlation between pMTG *alpha* and RT increased sharply from the start of the action observation (550–670 ms; p < 0.05–0.005) and peaked around the last quarter period (750 ms; p < 0.005) of the actor's pointing movement. Higher significant RT-correlated BA18 *alpha* activity (250–370 ms; p < 0.005–0.0001) preceded that of BA19 *alpha* activity (370–530 ms; p < 0.05–0.005), beginning almost in par with the onset of the actor's movement. We observed that the strength and significance of correlation between BA18 *alpha* and RT gradually increased with perceptual certainty with regard to eventual response cue information (650–1110 ms; p < 0.0001–0.00001) and prior to the actual hand response (1300 ms; p < 0.00001). In contrast, BA19 manifested a relatively sustained correlation with RT in its *alpha* modulation, which was highly significant from the middle (530–950 ms; p < 0.005) of the actor's pointing movement until participants made their response (1410 ms). Significant RT-correlated *beta* oscillatory processes emerged later towards the end of the actor's pointing movement in visual areas (BA18; 530–1170 ms, BA19; 570–1130 ms). The strength of RT-correlated PPC and PMd *beta* activity gradually built up and reached significance earlier for PPC (890 ms; p < 0.05) than PMd (970 ms; p < 0.05) in the interval prior to response cue onset.

Inline Supplementary Table S3

**Table S3:** Significant RT-related *alpha* and *beta* modulations within contrast-statistics derived ROIs. A. Significant associations between the frequency-specific lateralized power modulations within the paired ROIs and RT, as determined by the moving average correlation. These are listed by frequency of interest (foi); contrast-statistics defined ROI; on- and off-sets of significant associations (t_start, t_end); the corresponding correlation value (rho_start, rho_end); and the corresponding strength of association (p-value category: ns.; p < 0.05; p < 0.005; p < 0.0001; p < 0.00001). B. Same as in A. but sorted by onsets of significant associations. This provides the sequence of response-related associations during which different ROIs partake with varying prominence in their frequency-specific modulations. Both lists are color coded as those corresponding to ROI-specific moving average correlation plots in Fig. 5.

A									B								
	foi	ROI	t_start (s)	rho_start	pval_category		t_end (s)	rho_end		foi	ROI	t_start (s)	rho_start	pval_category		t_end (s)	rho_end
	**alpha**	BA6	na	na	n.s.		na	na		**alpha**	BA18	0.05	0.2927	0.05		0.25	0.4179
	**alpha**	BA39pMTG	0.55	0.2913	0.05		0.67	0.4154		**beta**	BA19	0.15	0.2869	0.05		0.19	0.287
	**alpha**	BA39pMTG	0.67	0.4154	0.005		0.85	0.4016		**alpha**	BA18	0.25	0.4179	0.005		0.37	0.5332
	**alpha**	BA39pMTG	0.75	0.4596	0.005	MAX				**alpha**	BA18	0.37	0.5332	0.0001		0.43	0.5323
	**alpha**	BA39pMTG	0.85	0.4016	0.005		0.97	0.2937		**alpha**	BA19	0.37	0.2874	0.05		0.53	0.3913
	**alpha**	BA18	0.05	0.2927	0.05		0.25	0.4179		**alpha**	BA18	0.43	0.5323	0.005		0.65	0.5323
	**alpha**	BA18	0.25	0.4179	0.005		0.37	0.5332		**alpha**	BA19	0.53	0.3913	0.005		1.41	0.4086
	**alpha**	BA18	0.37	0.5332	0.0001		0.43	0.5323		**beta**	BA18	0.53	0.2898	0.05		0.65	0.4048
	**alpha**	BA18	0.43	0.5323	0.005		0.65	0.5323		**alpha**	BA39pMTG	0.55	0.2913	0.05		0.67	0.4154
	**alpha**	BA18	0.65	0.5323	0.0001		1.11	0.5922		**beta**	BA19	0.57	0.2882	0.05		0.67	0.3925
	**alpha**	BA18	1.11	0.5922	0.00001		1.39	0.5952		**alpha**	BA18	0.65	0.5323	0.0001		1.11	0.5922
	**alpha**	BA18	1.29	0.614	0.00001	MAX				**beta**	BA18	0.65	0.4048	0.005		0.81	0.5384
	**alpha**	BA18	1.39	0.5952	0.00001		1.49	0.5428		**alpha**	BA39pMTG	0.67	0.4154	0.005		0.85	0.4016
	**alpha**	BA18	1.49	0.5428	0.005		1.55	0.4985		**beta**	BA19	0.67	0.3925	0.005		0.95	0.4028
	**alpha**	BA19	0.37	0.2874	0.05		0.53	0.3913		**alpha**	BA39pMTG	0.75	0.4596	0.005	MAX		
	**alpha**	BA19	0.53	0.3913	0.005		1.41	0.4086		**beta**	BA18	0.81	0.5384	0.0001		0.87	0.5379
	**alpha**	BA19	0.95	0.4898	0.005	MAX				**beta**	BA19	0.81	0.4829	0.005	MAX		
	**alpha**	BA19	1.41	0.4086	0.05		1.53	0.2846		**beta**	BA18	0.83	0.5426	0.0001	MAX		
	**alpha**	PPC	na	na	n.s.		na	na		**alpha**	BA39pMTG	0.85	0.4016	0.005		0.97	0.2937
	**beta**	BA6	0.97	0.2886	0.05		1.25	0.3027		**beta**	BA18	0.87	0.5379	0.005		1.03	0.3995
	**beta**	BA6	1.17	0.369	0.05	MAX				**beta**	PPC	0.89	0.2938	0.05		1.09	0.4008
	**beta**	BA39pMTG	na	na	n.s.		na	na		**alpha**	BA19	0.95	0.4898	0.005	MAX		
	**beta**	BA18	0.53	0.2898	0.05		0.65	0.4048		**beta**	BA19	0.95	0.4028	0.05		1.13	0.2856
	**beta**	BA18	0.65	0.4048	0.005		0.81	0.5384		**beta**	BA6	0.97	0.2886	0.05		1.25	0.3027
	**beta**	BA18	0.81	0.5384	0.0001		0.87	0.5379		**beta**	BA18	1.03	0.3995	0.05		1.17	0.2867
	**beta**	BA18	0.83	0.5426	0.0001	MAX				**beta**	PPC	1.09	0.4008	0.005		1.27	0.4045
	**beta**	BA18	0.87	0.5379	0.005		1.03	0.3995		**alpha**	BA18	1.11	0.5922	0.00001		1.39	0.5952
	**beta**	BA18	1.03	0.3995	0.05		1.17	0.2867		**beta**	BA6	1.17	0.369	0.05	MAX		
	**beta**	BA18	1.31	0.2855	0.05		1.43	0.4026		**beta**	BA19	1.17	0.2848	0.05		1.55	0.366
	**beta**	BA18	1.43	0.4026	0.005		1.55	0.4942		**beta**	PPC	1.21	0.4423	0.005	MAX		
	**beta**	BA19	0.15	0.2869	0.05		0.19	0.287		**beta**	PPC	1.27	0.4045	0.05		1.33	0.2947
	**beta**	BA19	0.57	0.2882	0.05		0.67	0.3925		**alpha**	BA18	1.29	0.614	0.00001	MAX		
	**beta**	BA19	0.67	0.3925	0.005		0.95	0.4028		**beta**	BA18	1.31	0.2855	0.05		1.43	0.4026
	**beta**	BA19	0.81	0.4829	0.005	MAX				**alpha**	BA18	1.39	0.5952	0.00001		1.49	0.5428
	**beta**	BA19	0.95	0.4028	0.05		1.13	0.2856		**alpha**	BA19	1.41	0.4086	0.05		1.53	0.2846
	**beta**	BA19	1.17	0.2848	0.05		1.55	0.366		**beta**	BA18	1.43	0.4026	0.005		1.55	0.4942
	**beta**	PPC	0.89	0.2938	0.05		1.09	0.4008		**alpha**	BA18	1.49	0.5428	0.005		1.55	0.4985
	**beta**	PPC	1.09	0.4008	0.005		1.27	0.4045		**alpha**	BA6	na	na	n.s.		na	na
	**beta**	PPC	1.21	0.4423	0.005	MAX				**alpha**	PPC	na	na	n.s.		na	na
	**beta**	PPC	1.27	0.4045	0.05		1.33	0.2947		**beta**	BA39pMTG	na	na	n.s.		na	na

Inline Supplementary Table S3

By sorting the relative onsets of these significant associations (see Inline Supplementary Table S3B), we can further appreciate the interplay of both *alpha* and *beta* oscillatory processes across the ROIs over the course of attentive-tracking and its influence on response preparation. This apparent interaction began within early visual areas (BA18 &BA 19), although mostly *alpha* activity dominated. Subsequently, pMTG *alpha* processes emerged while visual areas continued to be prominently involved. Decisively, the sequence of peak correlations beginning with *alpha* modulations within pMTG (750 ms), followed by *beta* modulations first in BA19 (810 ms) and then BA18 (830 ms) occurred prior the late manifestations of significant *beta* mediated associations in PPC (890 ms). RT-related *alpha* modulations in BA19 peaked (950 ms) prior to *beta* processes within BA6 (970 ms) manifesting significant association, which peaked just after response–cue onset (1170 ms). Thereafter, RT-correlated *beta* modulations within PPC peaked swiftly (1210 ms) followed by RT-related *alpha* modulations in BA18 (1290 ms) prior to participants' responses.

## Discussion

There is accumulating consensus that oscillatory activity in the brain functionally contributes to the allocation of attention in space (e.g., Rihs et al., 2009), to motor preparation and execution (e.g., Fujioka et al., 2012; Pogosyan et al., 2009; Tzagarakis et al., 2010), as well as to action observation (e.g., Kilner et al., 2009; Press et al., 2011). However, while brain regions involved in attentive tracking of dynamic stimuli have been previously investigated (Culham et al., 1998), the frequency-specific neural processes induced during and after action observation within sensory, attention-related, and motor regions of the brain have not been systematically or jointly investigated. We addressed this issue by using advanced MEG analyses techniques that allowed us to demonstrate the dynamic unfolding of a complex temporal pattern of (*alpha*- and *beta*-band) oscillatory activity within different brain regions. Notably, these oscillatory neural responses were related to on-going changes in both the spatial allocation of attention and the activation state within the motor cortex, which in turn were predictive of participants' overt performance. We summarize the present findings for *alpha*- and *beta*-band oscillations and their implications regarding their functional interpretation separately, before discussing how these frequency-specific processes are dynamically engaged during the observation of biological motion stimuli.

### Updating of spatial attention by incoming sensory evidence

We hypothesized that allocation of attention in space to different pointing movements (e.g., straight vs. crossed and/or left vs. right moving hand) is distinctively reflected in the online dynamics of the observers' neural modulations in both *alpha* and *beta* oscillations. The characteristic anticipatory activity of posterior *alpha* was evident very early on, in fact bilaterally, when it was equally uncertain as to which hemifield would be relevant for continued tracking before any discernible *Stimulus-Type* feature appeared. According to the view that *alpha* oscillations mirror an inhibitory process (Foxe and Snyder, 2011; Klimesch, 2012; Klimesch et al., 2007), the present stimulus-specific rebound following initial bilateral *alpha* oscillatory power decrease could reflect the active inhibition of information processing in the visual hemifield wherein the actor's arm remained stationary or started to move from its initial resting position. Specifically, decreased *alpha* oscillations in contralateral compared to ipsilateral PO areas represented the potential endpoint (i.e., target location) of the actor's hand movement. This effect is likely driven by the corresponding *alpha* power increase in the ipsilateral hemisphere (Kelly et al., 2006; Rihs et al., 2009), which endured until participants executed their response.

Based on these observations, we assume that the temporally evolving *alpha* power modulations reflect the observers' continuous extraction and prediction of the actor's movement endpoint from incoming dynamic stimulus information. Thus, incoming sensory information is constantly used to update predictions about the actor's movement endpoint (where the behaviorally relevant response cue would occur) and hence spatial certainty about the endpoint accumulates over time. Extending previous observations that attentional cues modulated anticipatory *alpha* activity depending on the degree of validity with which they indicated the location of the forthcoming target (Gould et al., 2011), we show that increasing spatial certainty during on-going attentive tracking modulates *alpha* activity. Certainly, motion energy differed between the actor's movement conditions (straight vs. crossed). That is, straight movements have more vertical motion relative to horizontal motion energy, while crossed movements would be predicted to exhibit stronger horizontal motion energies relative to the vertical. In principal, these differences in motion energy might have also induced the observed modulations of hand-specific *beta* lateralization. However, it seems unlikely that motion energy differences alone can account for the differential *alpha* modulations observed in our study for the following reason. If motion energy were to be responsible for the modulations in *alpha*, one would expect zero crossings in the lateralized oscillatory *alpha* activity for crossed pointing actions, because the actor's movement crosses observer's hemifields. However, such a pattern was clearly not observed in lateralized *alpha* oscillatory activity.

Together, our results indicate that lateralized parieto-occipital *alpha* oscillations sensitively reflect the changing allocation of attention dependent on the position of the actor's movement in space and the anticipated endpoint location. These findings advance previous reports that showed *alpha* activity to be a crucial substrate of visual input regulation (Romei et al., 2010) and to be actively involved in the deployment of spatial attention (Rihs et al., 2009; Thut et al., 2006) with reference to retinotopic coordinates (Worden et al., 2000). Specifically, our observations demonstrate that changes in on-going *alpha* activity relate to attentive tracking of dynamic stimuli rather than to the likely target position indicated by a symbolic precue presented at fixation, typical of research paradigms used in previous studies. Finally, an exciting possibility that deserves further investigation is that *alpha*-mediated allocation of spatial attention influences anticipatory response processes, as reflected by motor-related *beta* activity, before the actual response is indicated by the imperative response cue. We will discuss these motor-related oscillatory activity changes next.

### Priming of parieto-premotor areas by incoming sensory evidence

We assume that lateralized *beta* modulations reflect the observers' evolving response bias as a function of the actor's hand position in space. Thus, rather than the actor's moving effector, it was the visual hemifield within which the actor's hand was moving that determined the *beta* lateralization in motor areas, reflecting the dynamically changing response bias. Importantly, and as hypothesized, bias for a right hand response was reflected by stronger *beta* suppression for contralateral left motor areas as compared to ipsilateral right motor areas, and this resulted in faster RT if a right hand than a left hand response was required. Thus, the magnitude of this response bias (lateralized *beta*) predicted participants' RT; larger biases were associated with significantly faster responses, particularly those made in spatial congruence with movement endpoint location. In brief, *beta* lateralization power just before the onset of the response cue reliably reflects the response bias and predicts response time.

It is important to note that the response-related power modulation rode on top of the characteristic decrease of *beta* oscillatory activity that already began at stimulus onset. This power reduction manifested itself bilaterally and reflected a ‘general state of movement preparation’ (Pfurtscheller, 1981) for responding with either hand. This is in line with the overall task requirement that the precise response is only known upon presentation of the response cue, that is, at the very end of the actor's pointing movement. Since the required response is initially unknown, participants could refrain from activating a specific (left or right) response, guess and selectively activate one of the two responses, or simultaneously activate both potential responses (Jentzsch et al., 2004). In fact, and consistent with ERP findings of Jentzsch et al. (2004) in the response precuing paradigm, we observed an early and persistent bilateral *beta* oscillatory power decrease that we take to indicate parallel response activation. Moreover, with increasing spatial ‘certainty’ about the endpoint position, the spatially corresponding response becomes activated in the motor system, ultimately resulting in faster congruent than incongruent responses. Whereas ERP studies provided evidence for such location-based response priming following target onset (Stürmer et al., 2002), to our knowledge, this is the first study to demonstrate such a sensorimotor priming effect prior to response cue onset in *beta* oscillatory activity within parietal and premotor areas.

Finally, it is more commonly appreciated now that *beta* oscillatory activity is not solely motoric (Engel and Fries, 2010). For example, recent research showed that external entrainment of motor cortical activity at *beta* frequency (20 Hz) resulted in more slowly executed movements (Pogosyan et al., 2009) and a reduction in the number of unintended ‘no-go’ responses (Joundi et al., 2012). Existing studies have also shown that pre-response *beta* oscillatory activity is sensitive to experimental factors (Confais et al., 2012; Kilavik et al., 2012; Stančák et al., 1997; Tzagarakis et al., 2010), and could reflect predictive timing mechanisms (Fujioka et al., 2012; Saleh et al., 2010), or the degree of certainty in perceptual decision making (Donner et al., 2009). As such, it is also conceivable that *beta* modulations are influenced by *alpha*-mediated attention processes.

### The interplay of *alpha* and *beta* modulations

Looking at the time-course of oscillatory activity changes in different brain regions, the present study revealed some intriguing possibilities regarding the apparent interplay of *alpha*- and *beta*-band modulations. It is evident from the moving average correlations (100 ms; 50 ms resolution) between the frequency-specific lateralized ROI power modulations and behavioral response times that *alpha* and *beta* processes within visual (BA 18, BA 19), extrastriate (pMTG), parietal (BA 7; ILP) and premotor (PMd) areas participated in integrating relevant sensory information (i.e., spatial and response cues) during attentive tracking of the biological motion stimulus. Specifically, we observed that RT-related *alpha* oscillatory processes in visual brain areas BA 18, BA 19, and pMTG lead in the presumed accumulation of information (e.g., spatial likelihood to guide attention). The final contribution to action selection or activation appears to critically involve the emergence of stronger RT-related *beta* oscillatory processes within BA 18 and BA 19, just prior to that in the PPC (BA 7; BA 40), followed by PMd (BA 6), and the sustained and highly significant response-related *alpha* oscillatory processes in visual areas. The late onset of RT-correlated PMd mechanisms is consistent with the presumed active role this area plays in integrating incoming sensory and motor information from various brain sources (Pastor-Bernier and Cisek, 2011).

Crucially, the response-related frequency-specific signals within each ROI both emerged, and were also most prominently involved, at different time points during the sequence of action observation, cue onset, and response preparation. Of course, the proposed view regarding the interplay of *alpha*- and *beta*-band modulations in different brain regions should be considered preliminary. Nevertheless, as a working hypothesis, it can direct future research using analysis techniques that more directly assess cross-frequency signal interactions, such as phase-coupling analysis across different brain regions (cf. Siegel et al., 2008).

### Implications for the mechanisms involved in action observation

A final relevant aspect of the present research concerns the perhaps unsurprising finding that the brain areas significantly involved in our task overlap with those often reported in fMRI studies on action observation (Caspers et al., 2010; Rizzolatti and Fabbri-Destro, 2008; Rizzolatti et al., 2006), specifically the PMd (BA 6) and the PPC (BA 7; BA 40). However, it is worthwhile to note that in contrast to the present work, previous MEG studies investigating oscillatory activity during action observation generally reported mu-rhythm (8–12 Hz) and *beta* attenuation in the primary motor cortex (Caetano et al., 2007; Hari et al., 1998; Kilner et al., 2009). In addition, these studies were focusing on oscillatory activity during action observation within isolated brain regions (e.g., primary motor cortex). In this respect, it is remarkable that an EEG study of Babiloni et al. (2002) reported a decrease of *alpha* oscillatory power during the observation of movements at electrodes placed over parietal-occipital brain regions as well as *beta* oscillatory power reduction over motor areas. Certainly, since Babiloni et al. did not perform source-based analysis of oscillatory activity, their inferences concerning the brain sources involved in action observation processes remained vague. However, their findings are nevertheless similar to the present observation of cascaded oscillatory activity starting in visual regions (BA 18, BA 19), then extrastriate regions (pMTG), followed by the PPC (BA 7, BA 40), and then PMd (BA 6). This cascade of oscillatory changes is consistent with insights from neurophysiological studies, demonstrating overlapping neural activity within an integrated visuomotor processing network in the brain (Ledberg et al., 2007). Our observations also accord with the known cortico-cortical connectivity of the above brain regions and their postulated computations (Wise et al., 1997).

One final question then concerns the more precise functional role of the integrated oscillatory brain activity during action observation. Building on the initial insights of Babiloni et al., we hypothesize that dynamic stimulus information is first processed and motion information accumulated within visual areas BA 18, BA 19, and pMTG. We further assume that these areas continuously transmit processed information along the dorsal stream to the posterior parietal cortex (BA 7; BA 40). The PPC has been taken to play a pivotal role in sensorimotor integration (Andersen and Cui, 2009; Buneo and Andersen, 2006; Colby and Goldberg, 1999; Cui and Andersen, 2007; Gottlieb, 2007; Gottlieb and Balan, 2010) and in visually guided movements (Buneo and Andersen, 2006; Desmurget et al., 2009). More recently, the PPC has also been proposed to be central to the signaling of the intention to perform a certain action (Desmurget et al., 2009; Quian Quiroga et al., 2006). Crucially, the PMd is also important for the integration of visuomotor information (Pesaran et al., 2006), and ultimately plays a key part in coming up with a decision about the to-be-executed action (Cisek, 2006; Cisek and Kalaska, 2010). With this in mind, we speculate that during action observation, the PPC in concert with the PMd integrates incoming motion (or spatial) information from areas BA 18, BA 19, and pMTG with the observer's own action plans, thereby presumably facilitating simulation-based action understanding. However, as indicated by motor-related *beta* oscillatory changes during action observation, these simulations are not based on the moving limb of the actor (effector mirroring; Bertenthal et al., 2006; Koski et al., 2003) but rather related to the (dynamic) spatial position of the arm, consistent with Kilner et al.'s (2009) findings using a different action observation paradigm.

## Conclusions

Within the context of action observation, dynamic allocation of attention in space and the associated preparation of prospective response are reflected in observers' *alpha* and *beta* neural activity. Incoming sensory information can provide relevant salience cues that seize our attention, sometimes more than just momentarily, and influence our anticipatory gear-up for prospective action. Our findings suggest that amidst the parallel and sequential neural frequency processes, *beta* activity within parieto-frontal areas simultaneously participated in integrating *alpha*-mediated sensory salience and anticipatory response activation.

The following is the supplementary data related to this article.

Supplementary material.

## References

[bb0005] Andersen R.A., Cui H. (2009). Intention, action planning, and decision making in parietal–frontal circuits. Neuron.

[bb0010] Babiloni C., Babiloni F., Carducci F., Cincotti F., Cocozza G., Del Percio C., Moretti D.V., Rossini P.M. (2002). Human cortical electroencephalography (EEG) rhythms during the observation of simple aimless movements: a high-resolution EEG study. Neuroimage.

[bb0015] Bastiaansen M.C., Knosche T.R. (2000). Tangential derivative mapping of axial MEG applied to event-related desynchronization research. Clin. Neurophysiol..

[bb0020] Belopolsky A.V., Olivers C.N.L., Theeuwes J. (2008). To point a finger: attentional and motor consequences of observing pointing movements. Acta Psychol..

[bb0025] Bertenthal B.I., Longo M.R., Kosobud A. (2006). Imitative response tendencies following observation of intransitive actions. J. Exp. Psychol..

[bb0030] Brainard D.H. (1997). The psychophysics toolbox. Spat. Vis..

[bb0035] Buneo C.A., Andersen R.A. (2006). The posterior parietal cortex: sensorimotor interface for the planning and online control of visually guided movements. Neuropsychologia.

[bb0040] Caetano G., Jousmaki V., Hari R. (2007). Actor's and observer's primary motor cortices stabilize similarly after seen or heard motor actions. Proc. Natl. Acad. Sci..

[bb9085] Capilla A., Schoffelen J.M., Paterson G., Thut G., Gross J. (2012). Dissociated α-Band Modulations in the Dorsal and Ventral Visual Pathways in Visuospatial Attention and Perception. Cereb. Cortex.

[bb0045] Caspers S., Zilles K., Laird A.R., Eickhoff S.B. (2010). ALE meta-analysis of action observation and imitation in the human brain. Neuroimage.

[bb0050] Cisek P. (2006). Integrated neural processes for defining potential actions and deciding between them: a computational model. J. Neurosci..

[bb0055] Cisek P., Kalaska J.F. (2010). Neural mechanisms for interacting with a world full of action choices. Annu. Rev. Neurosci..

[bb0060] Colby C.L., Goldberg M.E. (1999). Space and attention in parietal cortex. Annu. Rev. Neurosci..

[bb0065] Confais J., Kilavik B.E., Ponce-Alvarez A., Riehle A. (2012). On the anticipatory precue activity in motor cortex. J. Neurosci..

[bb0070] Cui H., Andersen R.A. (2007). Posterior parietal cortex encodes autonomously selected motor plans. Neuron.

[bb0075] Culham J.C., Brandt S.A., Cavanagh P., Kanwisher N.G., Dale A.M., Tootell R.B.H. (1998). Cortical fMRI activation produced by attentive tracking of moving targets. J. Neurophysiol..

[bb0080] Desimone R. (1998). Visual attention mediated by biased competition in extrastriate visual cortex. Philos. Trans. R. Soc. B, Biol. Sci..

[bb0085] Desimone R., Duncan J. (1995). Neural mechanisms of selective visual attention. Annu. Rev. Neurosci..

[bb0090] Desmurget M., Reilly K.T., Richard N., Szathmari A., Mottolese C., Sirigu A. (2009). Movement intention after parietal cortex stimulation in humans. Science.

[bb0095] Donner T.H., Siegel M., Fries P., Engel A.K. (2009). Buildup of choice-predictive activity in human motor cortex during perceptual decision making. Curr. Biol..

[bb0100] Engel A.K., Fries P. (2010). Beta-band oscillations — signalling the status quo?. Curr. Opin. Neurobiol..

[bb0105] Foxe J.J., Snyder A.C. (2011). The role of alpha-band brain oscillations as a sensory suppression mechanism during selective attention. Front. Psychol..

[bb0110] Fujioka T., Trainor L.J., Large E.W., Ross B. (2012). Internalized timing of isochronous sounds is represented in neuromagnetic beta oscillations. J. Neurosci..

[bb0115] Gibson J.J. (1966). The Senses Considered as Perceptual Systems.

[bb0120] Gottlieb J. (2007). From thought to action: the parietal cortex as a bridge between perception, action, and cognition. Neuron.

[bb0125] Gottlieb J., Balan P. (2010). Attention as a decision in information space. Trends Cogn. Sci..

[bb0130] Gould I.C., Rushworth M.F., Nobre A.C. (2011). Indexing the graded allocation of visuospatial attention using anticipatory alpha oscillations. J. Neurophysiol..

[bb0135] Grèzes J., Tucker M., Armony J., Ellis R., Passingham R.E. (2003). Objects automatically potentiate action: an fMRI study of implicit processing. Eur. J. Neurosci..

[bb0140] Gross J., Kujala J., Hamalainen M., Timmermann L., Schnitzler A., Salmelin R. (2001). Dynamic imaging of coherent sources: Studying neural interactions in the human brain. Proc. Natl. Acad. Sci. U.S.A..

[bb0145] Händel B.F., Haarmeier T., Jensen O. (2010). Alpha oscillations correlate with the successful inhibition of unattended stimuli. J. Cogn. Neurosci..

[bb0150] Hanslmayr S., Aslan A., Staudigl T., Klimesch W., Herrmann C.S., Bäuml K. (2007). Prestimulus oscillations predict visual perception performance between and within subjects. Neuroimage.

[bb0155] Hari R., Forss N., Avikainen S., Kirveskari E., Salenius S., Rizzolatti G. (1998). Activation of human primary motor cortex during action observation: a neuromagnetic study. Proc. Natl. Acad. Sci. U.S.A..

[bb0160] Jensen O., Mazaheri A. (2010). Shaping functional architecture by oscillatory alpha activity: gating by inhibition. Front. Hum. Neurosci..

[bb0165] Jentzsch I., Leuthold H., Ridderinkhof K.R. (2004). Beneficial effects of ambiguous precues: parallel motor preparation or reduced premotoric processing time?. Psychophysiology.

[bb0170] Joundi R., Jenkinson N., Brittain J., Aziz T., Brown P. (2012). Driving oscillatory activity in the human cortex enhances motor performance. Curr. Biol..

[bb0175] Kelly S.P., Lalor E.C., Reilly R.B., Foxe J.J. (2006). Increases in alpha oscillatory power reflect an active retinotopic mechanism for distracter suppression during sustained visuospatial attention. J. Neurophysiol..

[bb0180] Kilavik B.E., Zaepffel M., Brovelli A., MacKay W.A., Riehle A. (2012). The ups and downs of beta oscillations in sensorimotor cortex. Exp. Neurol.

[bb0185] Kilner J.M. (2011). More than one pathway to action understanding. Trends Cogn. Sci..

[bb0190] Kilner J.M., Marchant J.L., Frith C.D. (2009). Relationship between activity in human primary motor cortex during action observation and the mirror neuron system. PLoS One.

[bb0195] Klimesch W. (2012). Alpha-band oscillations, attention, and controlled access to stored information. Trends Cogn. Sci..

[bb0200] Klimesch W., Sauseng P., Hanslmayr S. (2007). EEG alpha oscillations: the inhibition-timing hypothesis. Brain Res. Rev..

[bb0205] Koelewijn T., van Schie H.T., Bekkering H., Oostenveld R., Jensen O. (2008). Motor-cortical beta oscillations are modulated by correctness of observed action. Neuroimage.

[bb0210] Koski L., Iacoboni M., Dubeau M., Woods R.P., Mazziotta J.C. (2003). Modulation of cortical activity during different imitative behaviors. J. Neurophysiol..

[bb0215] Ledberg A., Bressler S.L., Ding M., Coppola R., Nakamura R. (2007). Large-scale visuomotor integration in the cerebral cortex. Cereb. Cortex.

[bb0220] Manly B.F.J. (2007). Chapter 8: regression analysis. Anonymous Randomization, Bootstrap and Monte Carlo Methods in Biology.

[bb0225] Oldfield R.C. (1971). The assessment and analysis of handedness: the Edinburgh inventory. Neuropsychologia.

[bb0230] Oostenveld R., Fries P., Maris E., Schoffelen J.M. (2011). FieldTrip: open source software for advanced analysis of MEG, EEG, and invasive electrophysiological data. Comput. Intell. Neurosci.

[bb0235] Pastor-Bernier A., Cisek P. (2011). Neural correlates of biased competition in premotor cortex. J. Neurosci..

[bb0240] Pelli D.G. (1997). The VideoToolbox software for visual psychophysics: transforming numbers into movies. Spat. Vis..

[bb0245] Pesaran B., Nelson M.J., Andersen R.A. (2006). Dorsal premotor neurons encode the relative position of the hand, eye, and goal during reach planning. Neuron.

[bb0250] Pfurtscheller G. (1981). Central beta rhythm during sensorimotor activities in man. Electroencephalogr. Clin. Neurophysiol..

[bb0255] Pogosyan A., Gaynor L.D., Eusebio A., Brown P. (2009). Boosting cortical activity at beta-band frequencies slows movement in humans. Curr. Biol..

[bb0260] Posner M.I., Snyder C.R., Davidson B.J. (1980). Attention and the detection of signals. J. Exp. Psychol. Gen..

[bb0265] Press C., Cook J., Blakemore S., Kilner J. (2011). Dynamic modulation of human motor activity when observing actions. J. Neurosci..

[bb0270] Quian Quiroga R., Snyder L.H., Batista A.P., Cui H., Andersen R.A. (2006). Movement intention is better predicted than attention in the posterior parietal cortex. J. Neurosci..

[bb0275] Rihs T.A., Michel C.M., Thut G. (2009). A bias for posterior α-band power suppression versus enhancement during shifting versus maintenance of spatial attention. Neuroimage.

[bb0280] Rizzolatti G., Fabbri-Destro M. (2008). The mirror system and its role in social cognition. Curr. Opin. Neurobiol..

[bb0285] Rizzolatti G., Fogassi L., Gallese V. (2006). Mirrors in the mind. Sci. Am..

[bb0295] Romei V., Rihs T., Brodbeck V., Thut G. (2008). Resting electroencephalogram alpha-power over posterior sites indexes baseline visual cortex excitability. Neuroreport.

[bb0300] Romei V., Brodbeck V., Michel C., Amedi A., Pascual-Leone A., Thut G. (2008). Spontaneous fluctuations in posterior α-band EEG activity reflect variability in excitability of human visual areas. Cereb. Cortex.

[bb0290] Romei V., Gross J., Thut G. (2010). On the role of prestimulus alpha rhythms over occipito-parietal areas in visual input regulation: correlation or causation?. J. Neurosci..

[bb0305] Saleh M., Reimer J., Penn R., Ojakangas C.L., Hatsopoulos N.G. (2010). Fast and slow oscillations in human primary motor cortex predict oncoming behaviorally relevant cues. Neuron.

[bb0310] Siegel M., Donner T.H., Oostenveld R., Fries P., Engel A.K. (2008). Neuronal synchronization along the dorsal visual pathway reflects the focus of spatial attention. Neuron.

[bb0315] Simon J.R. (1969). Reactions toward the source of stimulation. J. Exp. Psychol..

[bb0320] Stančák A., Riml A., Pfurtscheller G. (1997). The effects of external load on movement-related changes of the sensorimotor EEG rhythms. Electroencephalogr. Clin. Neurophysiol..

[bb0325] Stürmer B., Leuthold H., Soetens E., Schröter H., Sommer W. (2002). Control over location-based response activation in the Simon task: behavioral and electrophysiological evidence. J. Exp. Psychol..

[bb0330] Thompson J., Parasuraman R. (2012). Attention, biological motion, and action recognition. Neuroimage.

[bb0335] Thut G., Nietzel A., Brandt S.A., Pascual-Leone A. (2006). α-Band electroencephalographic activity over occipital cortex indexes visuospatial attention bias and predicts visual target detection. J. Neurosci..

[bb0340] Tzagarakis C., Ince N.F., Leuthold A.C., Pellizzer G. (2010). Beta-band activity during motor planning reflects response uncertainty. J. Neurosci..

[bb0345] van Dijk H., Schoffelen J., Oostenveld R., Jensen O. (2008). Prestimulus oscillatory activity in the alpha band predicts visual discrimination ability. J. Neurosci..

[bb0350] Van Veen B.D., Van Drongelen W., Yuchtman M., Suzuki A. (1997). Localization of brain electrical activity via linearly constrained minimum variance spatial filtering. IEEE Trans. Biomed. Eng..

[bb0355] Wise S.P., Boussaoud D., Johnson P.B., Caminiti R. (1997). Premotor and parietal cortex: corticocortical connectivity and combinatorial computations. Annu. Rev. Neurosci..

[bb0360] Worden M.S., Foxe J.J., Wang N., Simpson G.V. (2000). Anticipatory biasing of visuospatial attention indexed by retinotopically specific alpha-band electroencephalography increases over occipital cortex. J. Neurosci..

[bb0365] Wyart V., Tallon-Baudry C. (2009). How ongoing fluctuations in human visual cortex predict perceptual awareness: baseline shift versus decision bias. J. Neurosci..

