# Supplementary material for: Gearing up for action: Attentive tracking dynamically tunes sensory and motor oscillations in the alpha and beta band
Source: Neuroimage. 2013 Nov 15;82:634–44. doi: 10.1016/j.neuroimage.2013.04.120 (PMC3778976; doi:10.1016/j.neuroimage.2013.04.120)
Supplement: Inline Supplementary Table S1 [file mmc1.docx]

**Table S1.**

|  | **Main Effects** | **Abbrev.** | **Contrasts** | **Median RT (ms)**  **Mean ± SEM** | **Fstats**  F_(1,11)_ | ***p*** | **Sigf.** |
| --- | --- | --- | --- | --- | --- | --- | --- |
|  |  | | | | | | |
| i | *Actor’s Moving Hand* | *A* | Left | 450 ± 18 | 0.797 | 0.391 | n.s. |
|  |  |  | Right | 453 ± 18 |  |  |  |
|  |  | | | | | | |
| ii | *Cued Response Hand* | *R* | Left | 454 ± 19 | 0.476 | 0.505 | n.s. |
|  |  |  | Right | 448 ± 18 |  |  |  |
|  |  | | | | | | |
| Iii | *Endpoint Target Location* | *T* | Left | 453 ± 18 | 18.545 | 0.002 | ** |
|  |  |  | Right | 449 ± 18 |  |  |  |
|  |  | | | | | | |
| iv | INTERACTION | *A x R* | | | 4.898 | 0.049 | * |
|  |  | | | | | | |
| v | INTERACTION | *T x A* | | | 9.602 | 0.010 | * |
|  |  | | | | | | |
| vi | INTERACTION | *T x R* | | | 10.013 | 0.009 | ** |
|  |  | | | | | | |
| vii | INTERACTION | *A x T x R* | | | 5.073 | 0.046 | * |
|  |  | | | | | | |
| viii | *Stimulus-Type* | *S-type* | Straight | 455 ± 18 | 9.602 | 0.010 | * |
|  |  |  | Crossed | 448 ± 18 |  |  |  |
|  |  | | | | | | |
| ix | *Response-*Congruency | *R-congr.* | Congruent | 442 ± 19 | 10.013 | 0.009 | ** |
|  |  |  | Incongruent | 460 ± 18 |  |  |  |
|  |  | | | | | | |
| x | INTERACTION | *S-type x R-congr.* | | | 4.898 | 0.049 | * |
|  |  | | | | | | |

**Supplementary Table S1:** Summary of Analysis of Variance Analyses (ANOVA) assessing effects of salient factors on median response times (RT). 3-way ANOVA was performed with (i) *Actor’s Moving Hand*, (ii) *Cued Response Hand*, and (iii) *Endpoint Target Location* (abbreviated as *A, R, T*, respectively) as salient factors. 2-way ANOVA was performed with combined *Experimental-Conditions*: (viii) straight or crossed *Stimulus-Type* and (ix) *Response-Congruency* (abbreviated as *S-type* and *R-congr.*, respectively) as salient factors. Statistical significance is indicated by the asterisks: n.s. (non-significant); * (p<0.05); ** (p<0.005). Refer to text in the Results section and the Supplementary Results section for further details.
