# Supplementary material for: Gearing up for action: Attentive tracking dynamically tunes sensory and motor oscillations in the alpha and beta band
Source: Neuroimage. 2013 Nov 15;82:634–44. doi: 10.1016/j.neuroimage.2013.04.120 (PMC3778976; doi:10.1016/j.neuroimage.2013.04.120)
Supplement: Inline Supplementary Table S2 [file mmc2.docx]

**Table S2.**

|  | | | | | | | | | | |
| --- | --- | --- | --- | --- | --- | --- | --- | --- | --- | --- |
| **ROI#** | **MNI** | | | **Cerebral Hemisphere** | **Lobe** | **Landmark** | **Brodmann Area (BA)** | **Text Label** | **Hemispheric Regional MAX Voxel Density** | **Mean (FDR Stats)** |
|  | **X** | **Y** | **Z** |  |  |  |  |  |  |  |
|  |  |  |  |  |  |  |  |  |  |  |
| 1 | 18 | -18 | 64 | Right | Frontal | Precentral Gyrus | BA 6 | PMd | 4 | 2.79 |
| 2 | -18 | -18 | 64 | Left | Frontal | Precentral Gyrus | BA 6 |  |  |  |
| 3 | 54 | -64 | 8 | Right | Temporal | Middle Temporal Gyrus | BA 37 | pMTG | 6 | 4.35 |
|  |  |  |  |  |  |  | BA 39 |  |  |  |
| 4 | -54 | -64 | 8 | Left | Temporal | Middle Temporal Gyrus | BA 37 |  |  |  |
|  |  |  |  |  |  |  | BA 39 |  |  |  |
| 5 | 12 | -84 | 22 | Right | Occipital | Cuneus | BA 18 | BA 18 | 18 | 3.93 |
| 6 | -12 | -84 | 22 | Left | Occipital | Cuneus | BA 18 |  |  |  |
| 7 | 18 | -84 | 40 | Right | Parietal | Precuneus | BA 19 | BA 19 | 21 | 4.56 |
| 8 | -18 | -84 | 40 | Left | Parietal | Precuneus | BA 19 |  |  |  |
| 9 | 36 | -48 | 58 | Right | Parietal | Superior Parietal Lobule | BA 7 | PPC | 6 | 2.79 |
|  |  |  |  |  |  | Inferior Parietal Lobule | BA 40 |  |  |  |
| 10 | -36 | -48 | 58 | Left | Parietal | Superior Parietal Lobule | BA 7 |  |  |  |
|  |  |  |  |  |  | Inferior Parietal Lobule | BA 40 |  |  |  |

**Supplementary Table S2:** Statistically-determined task-relevant ROIs.

Bilateral ROIs were derived from the combined statistically significant *Stimulus-Type* and *Response-Congruency* contrasts’ regional maxima. MNI coordinates were used to find corresponding anatomical labels within the Fieldtrip toolbox (using the function *ft_prepare_atlas* which calls and accesses the AFNI brik file that is available from http://afni.nimh.nih.gov/afni/doc/misc/ttatlas_tlrc). For further details see “*Source-level analysis*” in Materials and methods section.
