# Supplementary material for: Gearing up for action: Attentive tracking dynamically tunes sensory and motor oscillations in the alpha and beta band
Source: Neuroimage. 2013 Nov 15;82:634–44. doi: 10.1016/j.neuroimage.2013.04.120 (PMC3778976; doi:10.1016/j.neuroimage.2013.04.120)
Supplement: Inline Supplementary Table S3 [file mmc3.docx]

**Table S3.**

| A |  |  |  |  |  |  |  |  |  | B |  |  |  |  |  |  |  |  |
| --- | --- | --- | --- | --- | --- | --- | --- | --- | --- | --- | --- | --- | --- | --- | --- | --- | --- | --- |
|  | **foi** | **ROI** | **t_start (s)** | **rho_start** | **pval_category** | | **t_end (s)** | **rho_end** |  |  | **foi** | **ROI** | **t_start (s)** | **rho_start** | **pval_category** | | **t_end (s)** | **rho_end** |
|  |  |  |  |  |  |  |  |  |  |  |  |  |  |  |  |  |  |  |
|  | **alpha** | BA6 | na | na | n.s. |  | na | na |  |  | **alpha** | BA18 | 0.05 | 0.2927 | 0.05 |  | 0.25 | 0.4179 |
|  | **alpha** | BA39pMTG | 0.55 | 0.2913 | 0.05 |  | 0.67 | 0.4154 |  |  | **beta** | BA19 | 0.15 | 0.2869 | 0.05 |  | 0.19 | 0.287 |
|  | **alpha** | BA39pMTG | 0.67 | 0.4154 | 0.005 |  | 0.85 | 0.4016 |  |  | **alpha** | BA18 | 0.25 | 0.4179 | 0.005 |  | 0.37 | 0.5332 |
|  | **alpha** | BA39pMTG | 0.75 | 0.4596 | 0.005 | MAX |  |  |  |  | **alpha** | BA18 | 0.37 | 0.5332 | 0.0001 |  | 0.43 | 0.5323 |
|  | **alpha** | BA39pMTG | 0.85 | 0.4016 | 0.005 |  | 0.97 | 0.2937 |  |  | **alpha** | BA19 | 0.37 | 0.2874 | 0.05 |  | 0.53 | 0.3913 |
|  | **alpha** | BA18 | 0.05 | 0.2927 | 0.05 |  | 0.25 | 0.4179 |  |  | **alpha** | BA18 | 0.43 | 0.5323 | 0.005 |  | 0.65 | 0.5323 |
|  | **alpha** | BA18 | 0.25 | 0.4179 | 0.005 |  | 0.37 | 0.5332 |  |  | **alpha** | BA19 | 0.53 | 0.3913 | 0.005 |  | 1.41 | 0.4086 |
|  | **alpha** | BA18 | 0.37 | 0.5332 | 0.0001 |  | 0.43 | 0.5323 |  |  | **beta** | BA18 | 0.53 | 0.2898 | 0.05 |  | 0.65 | 0.4048 |
|  | **alpha** | BA18 | 0.43 | 0.5323 | 0.005 |  | 0.65 | 0.5323 |  |  | **alpha** | BA39pMTG | 0.55 | 0.2913 | 0.05 |  | 0.67 | 0.4154 |
|  | **alpha** | BA18 | 0.65 | 0.5323 | 0.0001 |  | 1.11 | 0.5922 |  |  | **beta** | BA19 | 0.57 | 0.2882 | 0.05 |  | 0.67 | 0.3925 |
|  | **alpha** | BA18 | 1.11 | 0.5922 | 0.00001 |  | 1.39 | 0.5952 |  |  | **alpha** | BA18 | 0.65 | 0.5323 | 0.0001 |  | 1.11 | 0.5922 |
|  | **alpha** | BA18 | 1.29 | 0.614 | 0.00001 | MAX |  |  |  |  | **beta** | BA18 | 0.65 | 0.4048 | 0.005 |  | 0.81 | 0.5384 |
|  | **alpha** | BA18 | 1.39 | 0.5952 | 0.00001 |  | 1.49 | 0.5428 |  |  | **alpha** | BA39pMTG | 0.67 | 0.4154 | 0.005 |  | 0.85 | 0.4016 |
|  | **alpha** | BA18 | 1.49 | 0.5428 | 0.005 |  | 1.55 | 0.4985 |  |  | **beta** | BA19 | 0.67 | 0.3925 | 0.005 |  | 0.95 | 0.4028 |
|  | **alpha** | BA19 | 0.37 | 0.2874 | 0.05 |  | 0.53 | 0.3913 |  |  | **alpha** | BA39pMTG | 0.75 | 0.4596 | 0.005 | MAX |  |  |
|  | **alpha** | BA19 | 0.53 | 0.3913 | 0.005 |  | 1.41 | 0.4086 |  |  | **beta** | BA18 | 0.81 | 0.5384 | 0.0001 |  | 0.87 | 0.5379 |
|  | **alpha** | BA19 | 0.95 | 0.4898 | 0.005 | MAX |  |  |  |  | **beta** | BA19 | 0.81 | 0.4829 | 0.005 | MAX |  |  |
|  | **alpha** | BA19 | 1.41 | 0.4086 | 0.05 |  | 1.53 | 0.2846 |  |  | **beta** | BA18 | 0.83 | 0.5426 | 0.0001 | MAX |  |  |
|  | **alpha** | PPC | na | na | n.s. |  | na | na |  |  | **alpha** | BA39pMTG | 0.85 | 0.4016 | 0.005 |  | 0.97 | 0.2937 |
|  | **beta** | BA6 | 0.97 | 0.2886 | 0.05 |  | 1.25 | 0.3027 |  |  | **beta** | BA18 | 0.87 | 0.5379 | 0.005 |  | 1.03 | 0.3995 |
|  | **beta** | BA6 | 1.17 | 0.369 | 0.05 | MAX |  |  |  |  | **beta** | PPC | 0.89 | 0.2938 | 0.05 |  | 1.09 | 0.4008 |
|  | **beta** | BA39pMTG | na | na | n.s. |  | na | na |  |  | **alpha** | BA19 | 0.95 | 0.4898 | 0.005 | MAX |  |  |
|  | **beta** | BA18 | 0.53 | 0.2898 | 0.05 |  | 0.65 | 0.4048 |  |  | **beta** | BA19 | 0.95 | 0.4028 | 0.05 |  | 1.13 | 0.2856 |
|  | **beta** | BA18 | 0.65 | 0.4048 | 0.005 |  | 0.81 | 0.5384 |  |  | **beta** | BA6 | 0.97 | 0.2886 | 0.05 |  | 1.25 | 0.3027 |
|  | **beta** | BA18 | 0.81 | 0.5384 | 0.0001 |  | 0.87 | 0.5379 |  |  | **beta** | BA18 | 1.03 | 0.3995 | 0.05 |  | 1.17 | 0.2867 |
|  | **beta** | BA18 | 0.83 | 0.5426 | 0.0001 | MAX |  |  |  |  | **beta** | PPC | 1.09 | 0.4008 | 0.005 |  | 1.27 | 0.4045 |
|  | **beta** | BA18 | 0.87 | 0.5379 | 0.005 |  | 1.03 | 0.3995 |  |  | **alpha** | BA18 | 1.11 | 0.5922 | 0.00001 |  | 1.39 | 0.5952 |
|  | **beta** | BA18 | 1.03 | 0.3995 | 0.05 |  | 1.17 | 0.2867 |  |  | **beta** | BA6 | 1.17 | 0.369 | 0.05 | MAX |  |  |
|  | **beta** | BA18 | 1.31 | 0.2855 | 0.05 |  | 1.43 | 0.4026 |  |  | **beta** | BA19 | 1.17 | 0.2848 | 0.05 |  | 1.55 | 0.366 |
|  | **beta** | BA18 | 1.43 | 0.4026 | 0.005 |  | 1.55 | 0.4942 |  |  | **beta** | PPC | 1.21 | 0.4423 | 0.005 | MAX |  |  |
|  | **beta** | BA19 | 0.15 | 0.2869 | 0.05 |  | 0.19 | 0.287 |  |  | **beta** | PPC | 1.27 | 0.4045 | 0.05 |  | 1.33 | 0.2947 |
|  | **beta** | BA19 | 0.57 | 0.2882 | 0.05 |  | 0.67 | 0.3925 |  |  | **alpha** | BA18 | 1.29 | 0.614 | 0.00001 | MAX |  |  |
|  | **beta** | BA19 | 0.67 | 0.3925 | 0.005 |  | 0.95 | 0.4028 |  |  | **beta** | BA18 | 1.31 | 0.2855 | 0.05 |  | 1.43 | 0.4026 |
|  | **beta** | BA19 | 0.81 | 0.4829 | 0.005 | MAX |  |  |  |  | **alpha** | BA18 | 1.39 | 0.5952 | 0.00001 |  | 1.49 | 0.5428 |
|  | **beta** | BA19 | 0.95 | 0.4028 | 0.05 |  | 1.13 | 0.2856 |  |  | **alpha** | BA19 | 1.41 | 0.4086 | 0.05 |  | 1.53 | 0.2846 |
|  | **beta** | BA19 | 1.17 | 0.2848 | 0.05 |  | 1.55 | 0.366 |  |  | **beta** | BA18 | 1.43 | 0.4026 | 0.005 |  | 1.55 | 0.4942 |
|  | **beta** | PPC | 0.89 | 0.2938 | 0.05 |  | 1.09 | 0.4008 |  |  | **alpha** | BA18 | 1.49 | 0.5428 | 0.005 |  | 1.55 | 0.4985 |
|  | **beta** | PPC | 1.09 | 0.4008 | 0.005 |  | 1.27 | 0.4045 |  |  | **alpha** | BA6 | na | na | n.s. |  | na | na |
|  | **beta** | PPC | 1.21 | 0.4423 | 0.005 | MAX |  |  |  |  | **alpha** | PPC | na | na | n.s. |  | na | na |
|  | **beta** | PPC | 1.27 | 0.4045 | 0.05 |  | 1.33 | 0.2947 |  |  | **beta** | BA39pMTG | na | na | n.s. |  | na | na |
|  |  |  |  |  |  |  |  |  |  |  |  |  |  |  |  |  |  |  |

**Supplementary Table S3:**  Significant RT-related *alpha* and *beta* modulations within contrast-statistics derived ROIs

A. Significant associations between the frequency-specific lateralized power modulations within the paired ROIs and RT, as determined by the moving average correlation. These are listed by frequency of interest (foi); contrast-statistics defined ROI; on- and off-sets of significant associations (t_start, t_end); the corresponding correlation value (rho_start, rho_end); and the corresponding strength of association (p-value category: ns.; p<0.05; p<0.005; p<0.0001; p<0.00001). B. Same as in A. but sorted by onsets of significant associations. This provides the sequence of response-related associations during which different ROIs partake with varying prominence in their frequency-specific modulations. Both lists are color coded as those corresponding to ROI-specific moving average correlation plots in Fig.
