# Supplementary material for: Gearing up for action: Attentive tracking dynamically tunes sensory and motor oscillations in the alpha and beta band
Source: Neuroimage. 2013 Nov 15;82:634–44. doi: 10.1016/j.neuroimage.2013.04.120 (PMC3778976; doi:10.1016/j.neuroimage.2013.04.120)
Supplement: Supplementary file 4 — Supplementary material. [file mmc4.docx]

***Supplementary Material***

***Title:***

Gearing up for action: attentive tracking dynamically tunes sensory and motor oscillations in the *alpha* and *beta* band.

***Authors:***

Heng-Ru May Tan^1*^, Hartmut Leuthold ^2^, Joachim Gross^1^

***Author affiliation:***

^1^ Centre for Cognitive Neuroimaging (CCNi), Institute of Neuroscience and Psychology, College of Science and Engineering & College of Medical, Veterinary and Life Sciences, University of Glasgow, 58 Hillhead Street, Glasgow, G12 8QB, United Kingdom.

^2^ Department of Psychology, Eberhard Karls Universität Tübingen, Schleichstr. 4, 72076, Tübingen, Germany.

^*^ ***Corresponding Author:***

Heng-Ru May Tan (**Abbreviated reference name: Tan, H.-R.M.)

Centre for Cognitive Neuroimaging (CCNi), Institute of Neuroscience and Psychology, College of Science and Engineering & College of Medical, Veterinary and Life Sciences, University of Glasgow, 58 Hillhead Street, Glasgow, G12 8QB, United Kingdom.

Tel: +44 (0) 141 330 5090

E-mail: [Heng-RuMay.Tan@glasgow.ac.uk](mailto:Heng-RuMay.Tan@glasgow.ac.uk)

**Keywords:**

*alpha* and *beta* oscillations, dynamic stimuli, goal-directed behavior, magnetoencephalography (MEG), spatial attention, attentive tracking, action observation.

**Number of supplementary figures (2) and supplementary tables (3):** These are included in this document for ease of reference.

****High-resolution EPS figures and tables intended for inline referencing are provided on the online version of the manuscript (http://dx.doi.org/10.1016/j.neuroimage.2013.04.120).**

**Supplementary Methods**

***Selection of sensor clusters***

*Response-Type* related spectra neurodynamics were derived by averaging for each subject the relative power change spectra across trials which required (i) left, (ii) right, and (iii) no index finger responses (Supplementary Fig. S1A). We assessed the significance of *Response-Type* related changes in relative power spectra for each sensor. Contrasting the spectra neurodynamics for the condition that required a response (i or ii) against that for the non-response condition (iii) enabled the delineation of a temporal transition from perception-related to movement preparatory processes. Significant *t*-test critical T threshold (one-tailed at 95% level; *t*_(11)_ = 1.796) for *beta* frequency (24±2 Hz) indicated that the latter started around 250 ms after response cue onset (t = 1000 ms). This transitory time corresponded to the previously observed onset of *beta* oscillatory power rebound following no-go cues (Zhang *et al* 2008). From this transitory time point, two temporal ranges of interest were chosen to assist in sensor selection: a) perception-related interval (*toiP*) from 0 to +1250 ms relative to the stimulus onset, and b) movement-related interval (*toiM*) from +1250 to +1750 ms (Supplementary Figure S1).

Subsequently, we performed a one-sample dependent *t*-test against 0 for no relative change from baseline for the *Response-Type* related spectra neurodynamics. The one-sample *t*-values above the critical value (*t*_(11)_ = 2.2) were then separately averaged across a) *toiM* for trials corresponding to the aforementioned *Response-Type* related categories (i) and (ii), but across b) *toiP* for trials corresponding to *Response-Type* related category (iii). Thus, we derived an averaged *t*-value for both *alpha* (8-12 Hz) and *beta* (16-25 Hz) frequency bands, for each data category (i to iii), and each MEG sensor (N = 222).

For each of the averaged relative power change categories (i to iii) in the *beta* frequency, the MEG sensors associated with the highest 10 to 20 averaged *t*-values (where *t* > 2.2) were subsequently used to select four symmetric clusters of MEG sensors corresponding to Left Motor, Right Motor, Left Parieto-Occipital, Right Parieto-Occipital sensor-areas (Supplementary Fig. S1B). *Beta* frequency related *t*-values were preferentially used because the *alpha* frequency related *t*-values did not manifest clear lateralized effects for any of the data groups (i to iii). Each of the four clusters contained 12 MEG sensors. Subsequent analysis was performed on these hemisphere-specific modulations, that included a 300-ms baseline correction immediately before stimulus onset, for each of the eight response required *Experimental-Conditions* for *alpha* and *beta* frequency bands.

Reference

Zhang Y, Chen Y, Bressler SL, Ding M. 2008. Response preparation and inhibition: The role of the cortical sensorimotor beta rhythm. Neuroscience 156(1):238-246.

**Supplementary Results**

***Behavioral Median RTs***

Median RTs ranged from 300 to 610ms (Mean ± SEM = 451 ± 10ms) across subjects and stimulus-conditions. The three-way ANOVA of median RT (Supplementary Table S1) with repeated measures on factors: ($A$) *Actor’s Moving Hand*, ($T$) *Endpoint Target Location*, and ($R$) *Cued Response* on participants’ behavioral median RT revealed no significant main effects of the actor’s moving hand or of the cued response on RT (F_(1,11)_ = 0.797, p = 0.391; F_(1,11)_ = 0.476, p = 0.505, respectively). Responses were 4ms faster when endpoint target location was on the right than the left (F_(1,11)_ = 18.545, p = 0.002). The interaction between the actor’s moving hand and the cued response hand reached significance (F_(1,11)_ = 4.898, p = 0.049). More prominently, RT was significantly driven by the interaction between endpoint target location and actor’s moving hand (F_(1,11)_ = 9.602, p = 0.010), as well as the interaction between endpoint target location and cued responses (F_(1,11)_ = 10.013, p = 0.009). The three-way interaction among all three factors reached significance (F_(1,11)_ = 5.073, p = 0.046) but appears predominantly driven by endpoint target location.

The interaction between actor’s moving hand and target location revealed slightly faster responses if the endpoint target location (e.g. Left) and the actor’s moving hand (Left) matched compared to when they did not. Specifically, responses were about 7 ms faster when participants observed crossed than straight pointing movements, relative to the actor’s body midline. As expected, and as indicated by the interaction between endpoint target location and cued response hand, responses were faster when target location (e.g. Left) and cued response (e.g. Left) matched than when they did not, reflecting an effect of spatial *stimulus-response* congruence. The three-way interaction showed this *stimulus-response* congruence effect to be more pronounced for straight than crossed pointing movements.

***Brain source analysis***

Sensor-space regression analysis demonstrated significant *alpha* and *beta* modulations in a condition-specific manner particularly 600–1100ms post stimulus onset (Fig. 3A). We isolated bilateral brain areas involved in *alpha* and *beta* oscillatory processes associated with integrating the dynamic sensory information and action preparation during this time period (Supplementary Fig. S2). We found that viewing of straight pointing hand movements was associated with observer’s superior occipital and extra-striate (BA 19, pMTG) activity in the *alpha* frequency band, while visual areas (BA 18, BA 19) extending towards inferior parietal areas were significantly recruited in the *beta* band. Observation of crossed pointing movements yielded a weaker involvement in the visual areas, engaging mainly middle occipital cortex (BA 18) in the *alpha* band and sparse recruitment of the posterior parietal cortex (PPC; BA 7, BA 40) in the *beta* band.

The lateralized *alpha* power modulation manifested during congruent response trials was predominantly associated with occipital and extra-striate (BA 18, BA 19; pMTG) areas. In the *beta* band, trials requiring congruent responses significantly recruited the premotor area (PMd; BA 6) and occipital cortices. In the case of incongruent responses, posterior parietal areas (PCC) were recruited in addition to lateral-occipital visual areas (BA 18, BA 19) in the *alpha* frequency. Middle and superior occipital areas were mostly involved in the *beta* frequency in incongruent trials. Unlike its prominence in congruent trials, there was no significant involvement of premotor areas during incongruent trials, which typically took participants longer to respond (Fig. 2). This lends further support to the hypothesized response bias observed prior to response cue onset for congruent trials at the sensor-level analysis; anticipatory premotor activity significantly enhanced response speed in trials with congruent response cues.

**Supplementary Figure Legends**

**Supplementary Figure S1**

Selection of sensor clusters. (A) Grand-averaged time-frequency power modulations for trials requiring left, right, or no responses. The red vertical line separates the perception-related temporal range of interest (darker gray temporal bar; 0 to 1250 ms) and the movement-related temporal range of interest (lighter gray temporal bar; 1250 to 1750 ms relative to stimulus onset). (B) Selected clusters of sensors over left and right hemispheric Motor and Parieto-Occipital areas. Perception- and movement-related of MEG sensor clusters are indicated by light and dark gray shading, respectively.

**Supplementary Figure S2**

Significant brain sources related to *Stimulus-Type* and *Response-Congruency* in *alpha* and *beta* frequencies between 600 and 1100 ms after stimulus onset. Relative changes in time-frequency power modulations of derived brain sources were compared between hemispheres to validate lateralization effects. The color bar depicts FDR corrected *t*-statistic values corresponding to the significance of source contrast comparisons projected onto the brain surface. (Note: Depending on the conditional contrasts, e.g. *Stimulus-Type* or *Response-Congruency*, the “L minus R” contrast comparisons were made with respect to either the endpoint Target location or the Response hand, respectively. As such, the positive (red) and negative (blue) contrast *t*-statistics may appear flipped in some cases (i.e. incongruent response comparison; ~RL vs. ~LR) on the projected brain surface). The central brain surface plot depicts the combined significant brain sources and their regional absolute maxima were selected as regions of interest (ROIs; see “*Source-level analysis*” in Materials and methods section and Supplementary Table S1 for further details) in subsequent source-space analysis relating to response times. The brain surface plots are rendered by projecting sources maximally activated within 5 mm of brain volume, with projection threshold = 55% of the maximal *t*-statistics value.

**Fig. S1.**

**Fig. S2.**

**Table S1.**

|  | **Main Effects** | **Abbrev.** | **Contrasts** | **Median RT (ms)**  **Mean ± SEM** | **Fstats**  F_(1,11)_ | ***p*** | **Sigf.** |
| --- | --- | --- | --- | --- | --- | --- | --- |
|  |  | | | | | | |
| i | *Actor’s Moving Hand* | *A* | Left | 450 ± 18 | 0.797 | 0.391 | n.s. |
|  |  |  | Right | 453 ± 18 |  |  |  |
|  |  | | | | | | |
| ii | *Cued Response Hand* | *R* | Left | 454 ± 19 | 0.476 | 0.505 | n.s. |
|  |  |  | Right | 448 ± 18 |  |  |  |
|  |  | | | | | | |
| Iii | *Endpoint Target Location* | *T* | Left | 453 ± 18 | 18.545 | 0.002 | ** |
|  |  |  | Right | 449 ± 18 |  |  |  |
|  |  | | | | | | |
| iv | INTERACTION | *A x R* | | | 4.898 | 0.049 | * |
|  |  | | | | | | |
| v | INTERACTION | *T x A* | | | 9.602 | 0.010 | * |
|  |  | | | | | | |
| vi | INTERACTION | *T x R* | | | 10.013 | 0.009 | ** |
|  |  | | | | | | |
| vii | INTERACTION | *A x T x R* | | | 5.073 | 0.046 | * |
|  |  | | | | | | |
| viii | *Stimulus-Type* | *S-type* | Straight | 455 ± 18 | 9.602 | 0.010 | * |
|  |  |  | Crossed | 448 ± 18 |  |  |  |
|  |  | | | | | | |
| ix | *Response-*Congruency | *R-congr.* | Congruent | 442 ± 19 | 10.013 | 0.009 | ** |
|  |  |  | Incongruent | 460 ± 18 |  |  |  |
|  |  | | | | | | |
| x | INTERACTION | *S-type x R-congr.* | | | 4.898 | 0.049 | * |
|  |  | | | | | | |

**Supplementary Table S1:** Summary of Analysis of Variance Analyses (ANOVA) assessing effects of salient factors on median response times (RT). 3-way ANOVA was performed with (i) *Actor’s Moving Hand*, (ii) *Cued Response Hand*, and (iii) *Endpoint Target Location* (abbreviated as *A, R, T*, respectively) as salient factors. 2-way ANOVA was performed with combined *Experimental-Conditions*: (viii) straight or crossed *Stimulus-Type* and (ix) *Response-Congruency* (abbreviated as *S-type* and *R-congr.*, respectively) as salient factors. Statistical significance is indicated by the asterisks: n.s. (non-significant); * (p<0.05); ** (p<0.005). Refer to text in the Results section and the Supplementary Results section for further details.

**Table S2.**

|  | | | | | | | | | | |
| --- | --- | --- | --- | --- | --- | --- | --- | --- | --- | --- |
| **ROI#** | **MNI** | | | **Cerebral Hemisphere** | **Lobe** | **Landmark** | **Brodmann Area (BA)** | **Text Label** | **Hemispheric Regional MAX Voxel Density** | **Mean (FDR Stats)** |
|  | **X** | **Y** | **Z** |  |  |  |  |  |  |  |
|  |  |  |  |  |  |  |  |  |  |  |
| 1 | 18 | -18 | 64 | Right | Frontal | Precentral Gyrus | BA 6 | PMd | 4 | 2.79 |
| 2 | -18 | -18 | 64 | Left | Frontal | Precentral Gyrus | BA 6 |  |  |  |
| 3 | 54 | -64 | 8 | Right | Temporal | Middle Temporal Gyrus | BA 37 | pMTG | 6 | 4.35 |
|  |  |  |  |  |  |  | BA 39 |  |  |  |
| 4 | -54 | -64 | 8 | Left | Temporal | Middle Temporal Gyrus | BA 37 |  |  |  |
|  |  |  |  |  |  |  | BA 39 |  |  |  |
| 5 | 12 | -84 | 22 | Right | Occipital | Cuneus | BA 18 | BA 18 | 18 | 3.93 |
| 6 | -12 | -84 | 22 | Left | Occipital | Cuneus | BA 18 |  |  |  |
| 7 | 18 | -84 | 40 | Right | Parietal | Precuneus | BA 19 | BA 19 | 21 | 4.56 |
| 8 | -18 | -84 | 40 | Left | Parietal | Precuneus | BA 19 |  |  |  |
| 9 | 36 | -48 | 58 | Right | Parietal | Superior Parietal Lobule | BA 7 | PPC | 6 | 2.79 |
|  |  |  |  |  |  | Inferior Parietal Lobule | BA 40 |  |  |  |
| 10 | -36 | -48 | 58 | Left | Parietal | Superior Parietal Lobule | BA 7 |  |  |  |
|  |  |  |  |  |  | Inferior Parietal Lobule | BA 40 |  |  |  |

**Supplementary Table S2:** Statistically-determined task-relevant ROIs.

Bilateral ROIs were derived from the combined statistically significant *Stimulus-Type* and *Response-Congruency* contrasts’ regional maxima. MNI coordinates were used to find corresponding anatomical labels within the Fieldtrip toolbox (using the function *ft_prepare_atlas* which calls and accesses the AFNI brik file that is available from http://afni.nimh.nih.gov/afni/doc/misc/ttatlas_tlrc). For further details see “*Source-level analysis*” in Materials and methods section.

**Table S3.**

| A |  |  |  |  |  |  |  |  |  | B |  |  |  |  |  |  |  |  |
| --- | --- | --- | --- | --- | --- | --- | --- | --- | --- | --- | --- | --- | --- | --- | --- | --- | --- | --- |
|  | **foi** | **ROI** | **t_start (s)** | **rho_start** | **pval_category** | | **t_end (s)** | **rho_end** |  |  | **foi** | **ROI** | **t_start (s)** | **rho_start** | **pval_category** | | **t_end (s)** | **rho_end** |
|  |  |  |  |  |  |  |  |  |  |  |  |  |  |  |  |  |  |  |
|  | **alpha** | BA6 | na | na | n.s. |  | na | na |  |  | **alpha** | BA18 | 0.05 | 0.2927 | 0.05 |  | 0.25 | 0.4179 |
|  | **alpha** | BA39pMTG | 0.55 | 0.2913 | 0.05 |  | 0.67 | 0.4154 |  |  | **beta** | BA19 | 0.15 | 0.2869 | 0.05 |  | 0.19 | 0.287 |
|  | **alpha** | BA39pMTG | 0.67 | 0.4154 | 0.005 |  | 0.85 | 0.4016 |  |  | **alpha** | BA18 | 0.25 | 0.4179 | 0.005 |  | 0.37 | 0.5332 |
|  | **alpha** | BA39pMTG | 0.75 | 0.4596 | 0.005 | MAX |  |  |  |  | **alpha** | BA18 | 0.37 | 0.5332 | 0.0001 |  | 0.43 | 0.5323 |
|  | **alpha** | BA39pMTG | 0.85 | 0.4016 | 0.005 |  | 0.97 | 0.2937 |  |  | **alpha** | BA19 | 0.37 | 0.2874 | 0.05 |  | 0.53 | 0.3913 |
|  | **alpha** | BA18 | 0.05 | 0.2927 | 0.05 |  | 0.25 | 0.4179 |  |  | **alpha** | BA18 | 0.43 | 0.5323 | 0.005 |  | 0.65 | 0.5323 |
|  | **alpha** | BA18 | 0.25 | 0.4179 | 0.005 |  | 0.37 | 0.5332 |  |  | **alpha** | BA19 | 0.53 | 0.3913 | 0.005 |  | 1.41 | 0.4086 |
|  | **alpha** | BA18 | 0.37 | 0.5332 | 0.0001 |  | 0.43 | 0.5323 |  |  | **beta** | BA18 | 0.53 | 0.2898 | 0.05 |  | 0.65 | 0.4048 |
|  | **alpha** | BA18 | 0.43 | 0.5323 | 0.005 |  | 0.65 | 0.5323 |  |  | **alpha** | BA39pMTG | 0.55 | 0.2913 | 0.05 |  | 0.67 | 0.4154 |
|  | **alpha** | BA18 | 0.65 | 0.5323 | 0.0001 |  | 1.11 | 0.5922 |  |  | **beta** | BA19 | 0.57 | 0.2882 | 0.05 |  | 0.67 | 0.3925 |
|  | **alpha** | BA18 | 1.11 | 0.5922 | 0.00001 |  | 1.39 | 0.5952 |  |  | **alpha** | BA18 | 0.65 | 0.5323 | 0.0001 |  | 1.11 | 0.5922 |
|  | **alpha** | BA18 | 1.29 | 0.614 | 0.00001 | MAX |  |  |  |  | **beta** | BA18 | 0.65 | 0.4048 | 0.005 |  | 0.81 | 0.5384 |
|  | **alpha** | BA18 | 1.39 | 0.5952 | 0.00001 |  | 1.49 | 0.5428 |  |  | **alpha** | BA39pMTG | 0.67 | 0.4154 | 0.005 |  | 0.85 | 0.4016 |
|  | **alpha** | BA18 | 1.49 | 0.5428 | 0.005 |  | 1.55 | 0.4985 |  |  | **beta** | BA19 | 0.67 | 0.3925 | 0.005 |  | 0.95 | 0.4028 |
|  | **alpha** | BA19 | 0.37 | 0.2874 | 0.05 |  | 0.53 | 0.3913 |  |  | **alpha** | BA39pMTG | 0.75 | 0.4596 | 0.005 | MAX |  |  |
|  | **alpha** | BA19 | 0.53 | 0.3913 | 0.005 |  | 1.41 | 0.4086 |  |  | **beta** | BA18 | 0.81 | 0.5384 | 0.0001 |  | 0.87 | 0.5379 |
|  | **alpha** | BA19 | 0.95 | 0.4898 | 0.005 | MAX |  |  |  |  | **beta** | BA19 | 0.81 | 0.4829 | 0.005 | MAX |  |  |
|  | **alpha** | BA19 | 1.41 | 0.4086 | 0.05 |  | 1.53 | 0.2846 |  |  | **beta** | BA18 | 0.83 | 0.5426 | 0.0001 | MAX |  |  |
|  | **alpha** | PPC | na | na | n.s. |  | na | na |  |  | **alpha** | BA39pMTG | 0.85 | 0.4016 | 0.005 |  | 0.97 | 0.2937 |
|  | **beta** | BA6 | 0.97 | 0.2886 | 0.05 |  | 1.25 | 0.3027 |  |  | **beta** | BA18 | 0.87 | 0.5379 | 0.005 |  | 1.03 | 0.3995 |
|  | **beta** | BA6 | 1.17 | 0.369 | 0.05 | MAX |  |  |  |  | **beta** | PPC | 0.89 | 0.2938 | 0.05 |  | 1.09 | 0.4008 |
|  | **beta** | BA39pMTG | na | na | n.s. |  | na | na |  |  | **alpha** | BA19 | 0.95 | 0.4898 | 0.005 | MAX |  |  |
|  | **beta** | BA18 | 0.53 | 0.2898 | 0.05 |  | 0.65 | 0.4048 |  |  | **beta** | BA19 | 0.95 | 0.4028 | 0.05 |  | 1.13 | 0.2856 |
|  | **beta** | BA18 | 0.65 | 0.4048 | 0.005 |  | 0.81 | 0.5384 |  |  | **beta** | BA6 | 0.97 | 0.2886 | 0.05 |  | 1.25 | 0.3027 |
|  | **beta** | BA18 | 0.81 | 0.5384 | 0.0001 |  | 0.87 | 0.5379 |  |  | **beta** | BA18 | 1.03 | 0.3995 | 0.05 |  | 1.17 | 0.2867 |
|  | **beta** | BA18 | 0.83 | 0.5426 | 0.0001 | MAX |  |  |  |  | **beta** | PPC | 1.09 | 0.4008 | 0.005 |  | 1.27 | 0.4045 |
|  | **beta** | BA18 | 0.87 | 0.5379 | 0.005 |  | 1.03 | 0.3995 |  |  | **alpha** | BA18 | 1.11 | 0.5922 | 0.00001 |  | 1.39 | 0.5952 |
|  | **beta** | BA18 | 1.03 | 0.3995 | 0.05 |  | 1.17 | 0.2867 |  |  | **beta** | BA6 | 1.17 | 0.369 | 0.05 | MAX |  |  |
|  | **beta** | BA18 | 1.31 | 0.2855 | 0.05 |  | 1.43 | 0.4026 |  |  | **beta** | BA19 | 1.17 | 0.2848 | 0.05 |  | 1.55 | 0.366 |
|  | **beta** | BA18 | 1.43 | 0.4026 | 0.005 |  | 1.55 | 0.4942 |  |  | **beta** | PPC | 1.21 | 0.4423 | 0.005 | MAX |  |  |
|  | **beta** | BA19 | 0.15 | 0.2869 | 0.05 |  | 0.19 | 0.287 |  |  | **beta** | PPC | 1.27 | 0.4045 | 0.05 |  | 1.33 | 0.2947 |
|  | **beta** | BA19 | 0.57 | 0.2882 | 0.05 |  | 0.67 | 0.3925 |  |  | **alpha** | BA18 | 1.29 | 0.614 | 0.00001 | MAX |  |  |
|  | **beta** | BA19 | 0.67 | 0.3925 | 0.005 |  | 0.95 | 0.4028 |  |  | **beta** | BA18 | 1.31 | 0.2855 | 0.05 |  | 1.43 | 0.4026 |
|  | **beta** | BA19 | 0.81 | 0.4829 | 0.005 | MAX |  |  |  |  | **alpha** | BA18 | 1.39 | 0.5952 | 0.00001 |  | 1.49 | 0.5428 |
|  | **beta** | BA19 | 0.95 | 0.4028 | 0.05 |  | 1.13 | 0.2856 |  |  | **alpha** | BA19 | 1.41 | 0.4086 | 0.05 |  | 1.53 | 0.2846 |
|  | **beta** | BA19 | 1.17 | 0.2848 | 0.05 |  | 1.55 | 0.366 |  |  | **beta** | BA18 | 1.43 | 0.4026 | 0.005 |  | 1.55 | 0.4942 |
|  | **beta** | PPC | 0.89 | 0.2938 | 0.05 |  | 1.09 | 0.4008 |  |  | **alpha** | BA18 | 1.49 | 0.5428 | 0.005 |  | 1.55 | 0.4985 |
|  | **beta** | PPC | 1.09 | 0.4008 | 0.005 |  | 1.27 | 0.4045 |  |  | **alpha** | BA6 | na | na | n.s. |  | na | na |
|  | **beta** | PPC | 1.21 | 0.4423 | 0.005 | MAX |  |  |  |  | **alpha** | PPC | na | na | n.s. |  | na | na |
|  | **beta** | PPC | 1.27 | 0.4045 | 0.05 |  | 1.33 | 0.2947 |  |  | **beta** | BA39pMTG | na | na | n.s. |  | na | na |
|  |  |  |  |  |  |  |  |  |  |  |  |  |  |  |  |  |  |  |

**Supplementary Table S3:**  Significant RT-related *alpha* and *beta* modulations within contrast-statistics derived ROIs

A. Significant associations between the frequency-specific lateralized power modulations within the paired ROIs and RT, as determined by the moving average correlation. These are listed by frequency of interest (foi); contrast-statistics defined ROI; on- and off-sets of significant associations (t_start, t_end); the corresponding correlation value (rho_start, rho_end); and the corresponding strength of association (p-value category: ns.; p<0.05; p<0.005; p<0.0001; p<0.00001). B. Same as in A. but sorted by onsets of significant associations. This provides the sequence of response-related associations during which different ROIs partake with varying prominence in their frequency-specific modulations. Both lists are color coded as those corresponding to ROI-specific moving average correlation plots in Fig. 5.
